# An mRNA-based FimH nanoparticle vaccine against uropathogenic *Escherichia coli* confers protection in a mouse model of recurrent urinary tract infection

**DOI:** 10.3389/fimmu.2026.1920497

**Published:** 2026-09-03

**Authors:** Sandro Roier, Jessica M. Devant, Michael E. Hibbing, Annette Möbes, Carolin Kruth, Kristina Kovacikova, Steven M. Martin, Chanpheng Phommaly, Thomas J. Hannan, Edith Jasny

**Affiliations:** 1 CureVac SE, Tübingen, Germany; 2 Fimbrion Therapeutics Inc., Saint Louis, MO, United States

**Keywords:** fimH, mRNA vaccine, nanoparticle, UPEC, UPEC vaccine, urinary tract infection (UTI), uropathogenic *E. coli* (UPEC)

## Abstract

**Background:**

Increasing antimicrobial resistance and the high health and economic burden of frequent, recurring urinary tract infections (rUTIs) drive the need for an efficacious UTI vaccine. Most UTIs are caused by uropathogenic *E. coli* (UPEC) expressing the virulence factor FimH, which mediates bladder colonisation. We have developed a lipid nanoparticle (LNP) mRNA vaccine, FimH_DG_-Ferritin, as a UPEC vaccine candidate.

**Methods:**

FimH_DG_-Ferritin mRNA constructs were introduced into HeLa cells through mRNA-transfection to assess the generation of immune-accessible protein nanoparticles (PNPs) *in vitro* by native polyacrylamide gel electrophoresis and negative stain electron microscopy. Following this, induction of humoral and cellular responses were evaluated in female BALB/c AnNRj mice and Wistar rats following either 2 or 3 intramuscular administrations of the FimH_DG_-Ferritin mRNA vaccine. Finally, protective efficacy was assessed in a clinically relevant mouse model of rUTI.

**Results:**

*In vitro* PNP formation of the FimH_DG_-Ferritin fusion protein with intact functional epitopes was confirmed. Immunogenicity was demonstrated in mice and rats; equivalent functional antibody levels were raised against homologous and heterologous UPEC strains. In a rUTI mouse model, FimH_DG_-Ferritin mRNA vaccine administration induced the highest levels of functional antibodies among protein subunit comparators and significantly reduced bladder bacterial load versus negative controls.

**Conclusion:**

These preclinical data in rodents establish the FimH_DG_-Ferritin mRNA vaccine as a protective candidate vaccine to prevent UTI caused by UPEC, supporting consideration for phase 1 clinical evaluation.

## 1 Introduction

Urinary tract infections (UTIs) are one of the most common bacterial infections globally (1, 2). In their lifetime, at least half of all women are estimated to experience at least one symptomatic UTI (3, 4). A high proportion of antibiotic prescriptions in primary care settings are for UTI treatment, representing a significant public health burden (5). Despite antibiotic treatment, recurrent UTI (rUTI) – defined as two or more UTIs within 6 months, or three or more within a year – affects around one-third of women (3, 4). Most UTIs (~75%) are caused by uropathogenic *Escherichia coli* (UPEC) (6), in which antimicrobial resistance (AMR) is rising and exacerbating the burden of rUTI, particularly among women (7, 8). The high rate of rUTIs and increasing threat of AMR underscores the need for an effective UTI prevention strategy.

Targeted vaccination against bacterial strains that cause UTIs is a potential preventative approach that could reduce reliance on antibiotic treatment, helping to slow the spread of AMR (9). Whole cell and bacterial lysate vaccines against UPEC have been developed, namely Uro-Vaxom/OM-89, Solco-Urovac, and Uromune/MV140 (10–12); however, their long-term benefit has not been established (13), and no vaccine for the prevention of UTIs has received FDA approval to date (14). Following their successful development in response to the COVID-19 pandemic (15), mRNA vaccines against bacterial pathogens are now in early clinical development (16), offering advantages including favourable safety profiles, rapid and scalable production, and induction of strong humoral and cellular immune responses (17). Harnessing mRNA vaccine technology could be a promising alternative approach for developing a UTI vaccine.

The type 1 pilus is a critical UPEC virulence factor that mediates bacterial adhesion through the tip fibrillum lectin subunit FimH to mannosylated proteins (e.g., uroplakins) on the surface of bladder urothelial cells, allowing subsequent invasion of the bladder epithelium (18). The mannose-binding pocket of FimH is invariant among UPEC strains (19), with some residues outside of the pocket under strong positive selection in human clinical isolates (20). In mouse models, blocking FimH has therapeutic effects against UTI; antibodies that inhibit FimH binding to mannose confer protection through steric interference (21) and a high-affinity FimH antagonist can reduce both bladder and intestinal colonisation of diverse UPEC isolates (22–24). Therefore, FimH is a promising target in a UPEC vaccine strategy.

We have developed a lipid nanoparticle (LNP)-encapsulated mRNA vaccine with modified nucleosides for the prevention of UPEC-caused UTIs. The vaccine encodes the FimH protein in a pre-binding conformation (FimH_DG_) linked to *Helicobacter pylori* (*H. pylori*) ferritin, a protein nanoparticle (PNP)-forming domain designed to cluster FimH on the PNP surface (FimH_DG_-Ferritin) (25). The intrinsic stability and multimeric antigen presentation of ferritin particles is predicted to increase immunogenicity of vaccines harnessing this platform (26). Previous studies in rodents showed that administration of FimH_DG_-Ferritin mRNA vaccines induced higher functional antibody levels relative to a protein subunit vaccine control, and that multimeric presentation of FimH on ferritin PNPs elicited stronger immune responses than monomeric presentation (25).

In this study, we expanded our evaluation of the FimH_DG_-Ferritin mRNA vaccine candidate for the prevention of UTIs. We confirmed its ability to form FimH_DG_-Ferritin PNPs displaying copies of FimH suitable for functional antibody recognition. We evaluated the dose- and schedule-dependent immunogenicity of the FimH_DG_-Ferritin mRNA vaccine in mice and rats and assessed functional antibody responses against multiple UPEC strains. Finally, we demonstrated the protective efficacy of the vaccine in a clinically relevant mouse model of rUTI (27).

## 2 Materials and methods

### 2.1 FimH_DG_-Ferritin mRNA vaccine

The FimH_DG_-Ferritin mRNA vaccine has been described previously (25) and contained N1mΨ-modified nucleosides and comprised a 5’ cap1 structure, a 5’ untranslated region (UTR) from the human hydroxysteroid 17-beta dehydrogenase 4 gene (HSD17B4), a GC-enriched open reading frame (ORF), a 3’ UTR from the human proteasome 20S subunit beta 3 gene (PSMB3), a histone stem-loop, and a poly(A) tail. LNP encapsulation of mRNA was performed using LNP technology from Acuitas Therapeutics (Canada). LNPs were composed of an ionisable amino lipid, phospholipid, cholesterol, and a PEGylated lipid. The FimH_DG_-Ferritin mRNA vaccine-encoded FimH_DG_-fusion protein contains a signal peptide derived from human IgE (amino acid [aa] 1–18, NCBI reference sequence: AAB59424.1) at the N-terminus, followed by FimH from *E. coli* strain J96 (aa 22–300; NCBI reference sequence: ELL41155.1), a 5 aa linker (PGDGN), and the donor strand peptide of FimG from *E. coli* strain J96 (aa 24–37, NCBI reference sequence: ELL41154.1). A short SGG linker connects FimH_DG_ to *H. pylori* strain J99 Ferritin (aa 5–167; NCBI reference sequence: AAD06160.1) containing an N19Q mutation to remove a potential N-linked glycosylation site (28).

### 2.2 Cells

HeLa cells (DSMZ, Cat. ACC57), were derived from cervical adenocarcinoma and are of human origin. Cells were cultured in RPMI 1640 (Gibco, Cat. 52400-041) supplemented with 10% FCS (Gibco, Cat. 10270-106), 100 U/mL penicillin/100 mg/mL streptomycin (Gibco, Cat. 15140-122) and 2 mM L-glutamine (Gibco, Cat. 25030-024).

### 2.3 mRNA transfection

HeLa cells were seeded 24 hours (h) prior to transfection at a density of 1 x 10^6^ cells/flask (for transfection with LNP-formulated mRNA) or 2 x 10^6^ cells/flask (for Lipofectamine MessengerMax transfection (Invitrogen, Cat. LMRNA015). For the former, cells were transfected with 20 μg LNP-formulated mRNA per flask and incubated for 4 h before RPMI 1640 medium was replaced with Freestyle293 medium (Gibco, Cat. 12338-018). For the latter, RPMI 1640 medium was replaced with Opti-MEM (Gibco, Cat. 31985-070) medium prior to transfection with 40 μg mRNA per flask, using Lipofectamine MessengerMax according to manufacturer’s instructions. Briefly, mRNA and Lipofectamine MessengerMax were diluted in Opti-MEM medium and incubated for 10 minutes (min) at room temperature (RT). mRNA and Lipofectamine MessengerMax dilutions were combined, incubated for 5 min, and added onto the HeLa cell layer. After 90 min incubation, the medium was exchanged for Freestyle™293 medium. All transfected cells were collected after 72 h following a phosphate-buffered saline (PBS) wash step and lysed by addition of 2.5 mL native lysis buffer (50 mM Tris pH 7.4, 50 mM NaCl, 1mM EDTA, 1% DDM (n-Dodecyl-beta-D-maltoside), supplemented with protease inhibitor (Roche, Cat. A32955) for 30 min on ice. Collected lysate was cleared by centrifugation at 21300 RCF for 15 min at 4 °C.

### 2.4 FimH_DG_-Ferritin protein purification via Ni-NTA or mannose agarose beads

Transfected HeLa cell supernatant was harvested 72 h post transfection and centrifuged at 1500 RCF for 3 min at 4 °C to remove residual cells. Following the addition of protease inhibitor cocktail (Roche, Cat. A32955), the medium was incubated with Ni-NTA resin (Thermo Fisher Scientific, Cat. 88221) (for His-tagged FimH_DG_-Ferritin) or supernatant was batch-incubated with D-mannose agarose beads (Sigma-Aldrich, Cat. M6400) (for non-tagged FimH_DG_-Ferritin), overnight at 4 °C while shaking. The following day, the suspended resin was transferred to a gravity flow column. Unbound proteins were washed off with wash buffer (20 mM Tris, pH 7.4, 200 mM NaCl). For Ni-NTA purification, elution was performed with 6–8 column volumes of Ni-NTA elution buffer (20 mM Tris, pH 7.4, 200 mM NaCl, 250 mM imidazole). For mannose agarose beads, elution was performed with buffer containing increasing concentrations of α-methyl-mannopyranoside (2x elution with 5 mM,1x elution with 10 mM, 20 mM, 50 mM, 100 mM, 200 mM).

### 2.5 PNGase treatment

To remove glycans from FimH_DG_-Ferritin, the PNGase F kit (NEB, Cat. P0704) was used according to manufacturer’s instructions for denaturing reaction conditions. In brief, 1 μL of glycoprotein denaturing buffer was added to 9 μL of supernatant or lysate. The mix was denatured by boiling at 95 °C for 10 min. After cooling on ice, 2 μL Glyco Buffer 2, 2 μL NP-40, 6 μL of H_2_O and 1 μL of PNGase F was added. The reaction mixture was incubated at 37 °C for at least 1 h. Following the PNGase F treatment, samples were analysed via SDS-PAGE and western blot.

### 2.6 SDS-PAGE and western blot

Proteins were separated using SDS-PAGE. Lysates were denatured by mixing with loading dye (LI-COR, Cat. 928-40004) in 1:4 ratio and 10% β-mercaptoethanol (Sigma-Aldrich, Cat. M7522) before incubation at 95 °C for 5 min. The samples were loaded onto 4–15% Mini PROTEAN TGX gels (Bio-Rad, Cat. 4561086) and run in electrophoresis buffer (25 mM Tris, 192 mM glycine,0.1% SDS) at 150 V for 1 h. Following separation, gels were transferred onto a nitrocellulose membrane (LI-COR, Cat. 926-31092) in a wet-blotting setup for 1 h at 100 V. Blocking of the western blot membranes was performed with 5% milk powder in Tris-buffered saline (TBS) for 1 h at RT or overnight at 4 °C. Detection of proteins was performed using FimH-specific monoclonal antibody 54G9 (Fimbrion Therapeutics, St. Louis, USA).

### 2.7 Native PAGE and western blot

The oligomeric state of the expressed FimH_DG_-Ferritin protein was estimated using native PAGE (nPAGE) followed by western blot. Gels were run according to the manufacturer’s instructions (Bio-Rad) for native gels in native gel electrophoresis/blotting buffer (25 mM Tris, 192 mM glycine) at 200 V for 2–4 h. To estimate the size of the proteins, a native marker was used (Invitrogen, Cat. 57030). Following separation, gels were transferred onto a nitrocellulose membrane in a wet-blotting setup in the native gel electrophoresis/blotting buffer for 1 h at 100 V. For visualisation of the unstained native marker on the western blot, the membrane was stained with revert 520 total protein stain (Li-COR, Cat. 926-10011) and imaged prior to the blocking step on a Li-COR Odyssey M at 520 nm. Blocking of the western blot membranes was performed with 5% milk powder in TBS for 1 h at RT or overnight at 4 °C. Detection of proteins was performed using FimH-specific monoclonal antibody 54G9 (Fimbrion Therapeutics).

### 2.8 Gold labelling of antibody

To allow FimH-specific immunogold labelling on the electron microscopy (EM) grids, the FimH-specific monoclonal antibody 54G9 (Fimbrion Therapeutics) was covalently attached to 10 nm gold beads with a gold conjugation kit, according to manufacturer’s instructions (Abcam, Cat. 201808). Briefly, diluted antibody solution was mixed with the provided reaction buffer and added to the freeze-dried gold particles. After an incubation period of 15 min, the reaction was stopped by adding a quencher solution. The gold-conjugated antibody was stored at 4 °C.

### 2.9 Negative stain electron microscopy

For the directly stained grids, carbon foil was floated on water and subsequently transferred onto ultrathin carbon foil covered 3.5/1R Quantifoil grids. A 4 μL sample was applied to the grid and incubated for 1 min. The sample was removed using filter paper and washed 3 times with water. Subsequently, the grids were blotted and floated on a uranyl acetate solution for 1 min, before blotting with filter paper and air-drying. For the gold-conjugated antibody labelling of particles, 0.2 μL antibody was added to 30 μL sample. The sample mixture was incubated for 15 min on ice before preparing negative stain grids. Carbon Formvar grids (200 Mesh, Cu) were plasma cleaned and 4 μL sample was applied and incubated for 1 min. The grids were then blotted and washed 3 times with water. Subsequently, the grids were blotted and floated on a uranyl acetate solution for 1 min. Finally, the grids were blotted and air-dried.

### 2.10 Animal studies – immunogenicity of FimH_DG_-Ferritin mRNA vaccine in UPEC-naïve animals

#### 2.10.1 Ethics statement

UPEC-naïve female BALB/c AnNRj mice (total n=108) and Wistar IGS rats (total n=132) were obtained from Janvier Labs (France) and Charles River Laboratories (Sulzfield) arriving at 5 and 7 weeks of age, respectively. Animals were housed in Macrolon type III cages (n=10 mice/cage) or Macrolon type IV cages (n=3 rats/cage) and allowed access to food and water *ad libitum*. Animals were acclimatised for at least one week before any procedures were carried out. Animal rooms were kept temperature (19–23 °C) and humidity (35–65%) controlled with 12 h light/dark cycling and approximately 15 air changes per hour. To minimize potential confounding effects, animals were randomly assigned to treatment groups (n=12 animals/group), with group allocation balanced across cages and rack positions, and all animals were handled under standardized conditions. Studies were not blinded.

Immunogenicity studies were conducted by Preclinics Gesellschaft für präklinische Forschung mbH (GmbH), Germany in accordance with German laws and guidelines for animal care (directive 2010/63/EU). The studies were approved under the animal license number 2347-13-2023-10-G by the Brandenburg State Office for Occupational Safety, Consumer Protection and Health (LAVG).

Animals were visually inspected at least once daily for evidence of ill-health. After each injection, the injection sites were monitored daily for swelling, reddening, or other adverse effects until no deviations from the normal conditions could be detected. Body weight was measured at least once weekly throughout. Exclusion criteria were established *a priori.* Animals were euthanized upon reaching predefined humane endpoints, including ≥20% body weight loss from baseline or earlier if severe clinical signs were observed.

#### 2.10.2 mRNA vaccine preparation

The FimH_DG_-Ferritin mRNA vaccine was prepared for administration in BALB/c mice and Wistar rats by Preclinics Gesellschaft für präklinische Forschung mbH (Germany). All mRNA vaccine preparations were conducted under sterile and RNase-free conditions inside a laminar flow hood to prevent contamination and degradation of mRNA by RNases. Freshly opened sterile 0.9% NaCl solution (B. Braun Melsungen, Cat. 2350748) was used for dilution of the FimH_DG_-Ferritin mRNA vaccine. BD Micro-Fine 0.5 mL insulin syringes with 30 G x 8 mm needle (Embecta, Cat. 342825) were used for intramuscular (IM) injections.

#### 2.10.3 Immunisation

In mice, FimH_DG_-Ferritin mRNA vaccine was tested in 4 different doses ranging from 0.04 μg to 5 μg in a 2- or 3-dose immunisation schedule (n=12/group). In rats, 5 different doses ranging from 0.08 μg to 20 μg were assessed in 2- and 3-dose schedules (n=12/group). In both studies, animals receiving physiological saline (0.9% NaCl solution) served as a control (n=6/group). Due to the size of the rat study, animals were divided into 2 cohorts (n=12/cohort per vaccine group or n=6/cohort for 0.9% NaCl control group), and the dosing was initiated on 2 consecutive days. In mice, all mRNA vaccines were administered IM in a volume of 20 μL into the *Musculus tibialis* or in rats in a volume of 100 μL into the *Musculus gastrocnemius* using single-use insulin syringes with an integrated 30G needle (Embecta, Cat. 342825). Negative control animals received 0.9% NaCl solution (20 µL for mice; 100 µL for rats). The left and right hind leg were alternated in subsequent doses. Injections were performed on Days 0, 28 (2-dose schedule) or Days 0, 28 and 56 (3-dose schedule) following anaesthesia by inhalation of isoflurane (5 vol. %). Daily observations and clinical signs were documented, and animal body weight, injection site, and general physical condition was monitored throughout the study.

#### 2.10.4 Blood sampling

Intermediate blood samples were obtained by retro-orbital bleeding on Day 1 (18 h after first administration) and Day 28/29 for all groups in mice/rats. For blood collection, animals were anaesthetised using isoflurane (5 vol.%) and collection was performed using non-heparinised capillary (Minicaps 10 μL, VWR International GmbH, Cat.612-2439). For mice, 140 μL blood was collected into Z-clot activator 200 μL microtube (Sarstedt, Cat. 20.1291) and for rats, ~1.2 mL blood was collected into Z-clot activator 1.1 mL microtube (Sarstedt, Cat. 41.1500.005), incubated at RT for 30 min and centrifuged for 5 min, 10000 RCF, RT.

On Day 42 (2-dose schedule) or Day 70 (3-dose schedule), animals were sacrificed by exsanguination. Animals were anaesthetised with 5 vol. % isoflurane and the whole blood was removed by retro-orbital bleeding for mice, or by heart puncture in rats. Blood samples were incubated for 30 min at RT before centrifugation for 5 min, 10000 RCF, RT.

#### 2.10.5 Urine sampling

Spontaneous urine was collected from mice on Days 27 and 28 (all groups), 41 (2-dose schedule) and 69 (3-dose schedule). In rats, spontaneous urine was collected on Days 28 and 29 (all groups), 41 (2-dose schedule) and 69 (3-dose schedule). For this procedure, animals were placed in a type I cage with hydro-repellent sand bedding. After urine sampling, animals were placed back in their home cages. Urine collected on two consecutive days was pooled. If ≥100 μL (mice) or ≥200 μL (rats) of urine was collected on the first collection day, the sampling on the consecutive day was omitted. At the respective study endpoints for each dose schedules (Day 42 or Day 70), urine was collected from animals by bladder puncture, centrifuged at 3500 RCF at RT for 5 min, and supernatant was stored at –80 °C.

#### 2.10.6 Splenocyte isolation

Splenocytes were isolated by mechanical disruption of the spleen using GentleMACS (Miltenyi Biotec) adapted protocol. Red blood cells (RBCs) were lysed in 10 mL lysis buffer (144 mM ammonium chloride, Chemosolute, Cat. 2668.1000; 17mM Tris, Carl ROTH, Cat. 4855.1) centrifuged for 3 min at 453 RCF at RT, and splenocytes were washed, resuspended in foetal calf serum (FCS) with 10% DMSO and stored as single-cell suspensions in liquid nitrogen until use.

### 2.11 Animal studies – protective efficacy of the FimH_DG_-Ferritin mRNA vaccine in a rUTI mouse model

#### 2.11.1 Ethics statement

UPEC-naïve female C3H/HeN mice were obtained from Envigo, (Indianapolis, USA), arriving 6–8 weeks of age. Animals were housed in exhaust ducted OptiMouse caging (n=5 mice/cage) and allowed access to food and water *ad libitum*. Animals were acclimatised for at least one week before any procedures were carried out. Animal rooms were temperature (20–27 °C) and humidity (30–70%) controlled with 12 h light/dark cycling and 10.7 air changes per hour. To minimize potential confounding effects, animals were randomly assigned to treatment groups (detailed below), with group allocation balanced across cages and rack positions, and all animals were handled under standardized conditions. Studies were not blinded.

Protective efficacy experiments were performed by Fimbrion Therapeutics personnel. All procedures were performed in accordance with protocols approved by the Saint Louis University Institutional Animal Care and Use Committee (Protocol number: Hannan, #2633) and met or exceeded the standards of the American Association for the Accreditation of Laboratory Animal Care (AAALAC), the United States Department of Health and Human Services and all local and federal animal welfare laws.

Animals were monitored at least 2–3 times weekly for signs of pain or distress by facility staff and the day after immunisation. Potential signs of pain or distress included decreased activity, piloerection, ungroomed appearance, abnormal posture, and changes in respiration. Any mouse that lost >20% of their peak body mass or were observed to be acutely moribund (e.g. showed immobility, depression, rough coat) were euthanised. IACUC Guideline 002, Humane Endpoints were adhered to, and a facility veterinarian was available for consultation as needed. Exclusion criteria were established *a priori* and are further detailed below.

#### 2.11.2 Recurrent cystitis infection model

An initial infection was established in 172 female C3H/HeN mice using a superinfection model (29) to maximise the number of mice that would develop chronic cystitis, which is necessary for rUTI sensitisation. For this superinfection, 2 transurethral inoculations of UPEC into the urinary bladder were administered, 1 h apart. For these infections (30), mice were anaesthetised by inhalation of 3% isofluorane in 2.5 L/min of oxygen and 50 μL of prepared UPEC bacterial suspension (2–4 × 10^9^ colony forming units [CFUs]/mL) was inoculated into the bladder of each mouse using a transurethral catheter to dispense approximately 1–2 × 10^8^ CFUs/mouse. A second inoculum was prepared and administered 1 h after the initial infection. Infections were monitored for 2 weeks by quantification of urine bacterial burdens (at 3-, 7-, 10-, and 13-days post infection). Mice that maintained persistent bacteriuria of ≥10^6^ CFUs/mL urine were used for further analysis; the remaining mice were culled from the experiment. Mice maintaining persistent bacteriuria were treated with sulfamethoxazole-trimethoprim *ad libitum* in their drinking water (266.8 mg/53.4 mg/L water) for 10 days, beginning 14 days post infection. The mice were then returned to normal drinking water and allowed to convalesce for an additional 18 days. Urine bacterial titres were monitored during this time to detect potential kidney infections. Mice were excluded from the study if their urine samples post antibiotic treatment had: 1) one or more individual urine titres ≥10^6^ CFUs/mL; or 2) persistent bacteriuria ≥10^4^ CFUs/mL on two separate sampling days. Following sensitisation and convalescence, animals were weighed and randomly allocated to treatment groups, with the groups normalised by animal weight, using the program RandoMice v1.1.7. Overall, 154 of 172 mice (90%) were randomised into 12 treatment groups prior to immunisation. During the vaccination and pre-challenge phases, additional mice were excluded due to bacteriuria, resulting in final total of 147 mice suitable for analysis after challenge infection (8–13 animals per treatment group).

#### 2.11.3 mRNA and FimHC vaccine preparation

The FimH_DG_-Ferritin mRNA vaccine was tested along with negative and vaccine controls. The negative control was an irrelevant mRNA vaccine encoding the rabies virus glycoprotein (RABV-G; aa 1−524; NCBI reference sequence: AAA47218.1) and contained the same N1mΨ-modified nucleosides, mRNA backbone (GC enrichment, cap, 5’ and 3’ UTRs, histone stem-loop, and poly(A) tail) and LNP-formulation as the FimH_DG_-Ferritin mRNA vaccine. The vaccine controls were composed of a protein complex consisting of FimH and FimC (FimHC), adjuvanted with either Freund’s adjuvant (FimHC-Freund’s) or PHAD (FimHC-PHAD). FimHC, originally cloned from UPEC strain UTI89, was prepared at a concentration of 1.881 mg/mL, according to Schwartz et al. (20). Briefly, UTI89 FimH and FimC proteins were co-expressed in *Escherichia coli* C600 with a pBAD33 plasmid containing the UTI89 *fimH* allele and a pTRC99a plasmid containing UTI89 *fimC* allele. FimHC proteins complexes were isolated from crude periplasmic preparations using affinity chromatography and cleaned on an additional Source 15S column in 15mM 2-(N-morpholino) ethanesulfonic acid (pH 5.8) in the absence of mannose.

The concentrated mRNA vaccine formulations were diluted with 0.9% NaCl to final concentrations of 250 μg/mL (5 μg/dose) for the irrelevant mRNA vaccine and 250 and 10 μg/mL for the FimH_DG_-Ferritin mRNA vaccine (5 and 0.2 μg/dose, respectively). The FimHC-PHAD vaccine was prepared as described elsewhere (31) by combining FimHC and PHAD adjuvant to final concentrations of 750 and 200 μg/mL in PBS. The FimHC-Freund’s vaccine was prepared by diluting the FimHC protein complex with PBS to a concentration of 600 μg/mL, then emulsifying that solution with an equal volume of Freund’s complete adjuvant (FCA, Thermo Scientific, Cat. 77140) for the initial vaccination or Freund’s incomplete adjuvant (FIA, Thermo Scientific, Cat. 77145) for the additional doses. The FimHC/Freund’s emulsions were prepared using the Minilys personal homogeniser and the Precellys emulsion kit following manufacturer’s instructions (Bertin technologies, Cat. NC2065743).

The 0.9% NaCl solution, mRNA, and PHAD-adjuvanted vaccines were delivered by IM injection of 20 μL into the *Musculus tibialis anterior* using single-use syringes (BD Micro-Fine U100 0.5 mL insulin syringe with 30G x 8mm needle; Embecta, Cat. 324825). The Freund’s adjuvanted vaccines were delivered by subcutaneous injection (SQ) of 50 μL under the lateral thigh skin through a 25 G needle attached to a 1 mL insulin syringe. Vaccines for the 2-dose schedule were administered 42- and 70-days post infection while, due to study size, vaccines for the 3-dose schedule were administered 43-, 71-, and 98-days post infection.

#### 2.11.4 Serum sampling

To monitor binding and functional anti-FimH antibody levels in the serum, mice were anaesthetised, as described in the recurrent cystitis infection model section above, and blood samples were collected by submandibular vein puncture, using a 4-mm lancet, into serum separator tubes (BD Biosciences, Cat. 365976). Blood samples were incubated at RT for at least 30 min then centrifuged at 9503 RCF at 4 °C for 5 min to separate the serum. Serum samples were stored in the separator tubes at -20 °C for future analysis by enzyme-linked immunosorbent assay (ELISA) and haemagglutination inhibition (HAI) assay. For the 2-dose schedule mice, blood samples were collected at 84- and 99-days post initial infection. For the 3-dose schedule mice, blood samples were collected at 112- and 127-days post initial infection.

#### 2.11.5 Urine sampling

To monitor recalcitrant UTIs, assess anti-FimH antibody levels, and determine pre-challenge bacteriuria and post challenge cytokine induction, urine samples were collected the one day pre- and one day post-challenge from conscious mice by the application of gentle suprapubic pressure, collecting the expressed urine into sterile 1.5 mL tubes. After removing 10 μL of urine for bacterial enumeration, urine samples (ideally 50–100 μL) were centrifuged at RT at 16060 RCF for 2 min and supernatants transferred to fresh 1.5 mL tubes. Urine supernatants were stored at -20 °C. For the 2-dose schedule mice, urine samples were collected 97- and 99-days post initial infection. For the 3-dose schedule mice, urine samples were collected 125- and 127-days post initial infection.

#### 2.11.6 Challenge infection

Challenge infections were established by a single infection with 10^8^ CFUs of UTI89 prepared and administered transurethrally at 98-days post initial infection (2-dose schedule mice) or 126-days post initial infection (3-dose schedule mice). The reference clinical UPEC strain used in this study was UTI89 (27), which was isolated from a woman with cystitis. To prepare the UPEC strains for inoculation in mice, an aliquot from the freezer stock was added to 20 mL of Luria-Bertani (LB) broth and grown statically at 37 °C overnight. The overnight culture was diluted into fresh media by adding 30 μL into 30 mL of LB broth and subculturing statically at 37 °C for further 18 h. The cultures were centrifuged at RT for 10 min at 3000 RCF, resuspended in 10 mL PBS (pH 7.4), and diluted to approximately 2–4 × 10^9^ CFUs/mL by estimation using optical density (OD_600_ ~ 3.5) to prepare the final suspension for the infection procedure. The final CFUs/mL of the inoculum was confirmed by dilution plating.

#### 2.11.7 Tissue collection and determination of bacterial burden

To assess bacterial burdens and immune responses in the urinary tracts of challenged mice at 1-day post challenge (99-days post initial infection for the 2-dose schedule mice and 127-days post initial infection for the 3-dose schedule mice), urine and serum were collected from each mouse as described above 23–24 h post-challenge infection. Following blood and urine collection, mice were euthanised by isoflurane overdose followed by cervical dislocation. Bladders were aseptically removed and separately homogenised using a FastPrep24 bead homogeniser (MP Biomedicals) in PBS (1 mL final volume of tissue and buffer) in 2.0 mL screw cap conical tubes (Fisher Scientific, Cat. 02-707-355). Bladder homogenates and urine samples were then serially diluted in a 10-fold dilution series in PBS and plated in 5 spots (2 spots for urine) of 10 μL each onto LB agar plates. The remaining bladder homogenate samples were centrifuged at RT at 16060 RCF (max speed) for 5 min and supernatants transferred to fresh 1.5 mL tubes. Bladder homogenate supernatants were stored at -20 °C for cytokine analysis by ELISA. Following overnight incubation at 37 °C, the lowest dilution row that contained between 15 and 150 colonies was counted for quantification of bacterial burden. The lower limit of quantification was 1.3 log_10_ CFUs per bladder. Bacterial burden is presented as CFU/bladder (i.e, not normalised to bladder weight) to account for the relative homogeneity of bladder weights prior to infection and the immune response-driven heterogenicity of bladder weights post infection, previously demonstrated in this model (32). Normalisation to bladder weight could therefore introduce bias and was not performed.

### 2.12 Animal studies – immunological analysis

#### 2.12.1 Determination of binding antibody titres in sera and urine by ELISA

Quantities of FimH-specific binding antibodies (total IgG) in serum and urine samples of immunised animals were assessed by indirect ELISA. 96-well MaxiSorp ELISA plates were coated for 4–5 h at 37 °C with 1 μg/mL of a recombinant FimH protein (Biomatik, Cat. RPC25919) for serum and urine analysis, or with 1 μg/mL recombinant *H. pylori* ferritin (Cusabio, Cat. CSB-YP009053HUV),1 μg/mL recombinant human ferritin light chain (Sino Biological, Cat. 12482-H07E), or 5 μg/mL recombinant human ferritin heavy chain (Sino Biological, Cat. 13217-HNAE) for serum analysis. Plates were washed and blocked overnight at 4 °C with 5% milk in PBS/0.05% Tween-20 for mouse samples and 1% bovine serum albumin (BSA) in PBS/0.05% Tween-20 for rat samples. Serum samples were added to the test plates, serially diluted, and incubated for 2 h at RT. For urine sample preparation from the immunogenicity studies, the pH value of each urine sample was measured using a SevenDirect SD20 pH-device with an InLab Ultra-Micro-ISM pH electrode (Mettler Toledo). If the pH value was <7, urine samples were neutralised by mixing the samples 1:1 with PBS pH 8.2. If the pH value was ≥7, urine samples were directly diluted 1:2 in blocking buffer. Due to sample volume limitations in the protective efficacy study, urine samples were assumed to have pH <7 and were diluted and neutralised by a 1:4 dilution with PBS (pH 8.2) then further diluted 1:2 with blocking buffer (1:8 final initial dilution). Initial dilutions in PBS (pH 8.2) and/or blocking buffer were performed directly as a predilution before urine samples were added in serial dilution and incubated for 2 h at RT. After washing, plates were incubated either with HRP-conjugated goat anti mouse IgG (Jackson Immuno Research, Cat. 115-035-003, 1:5000) or HRP-conjugated goat anti rat IgG (Invitrogen, Cat. 31470, 1:10000) in blocking buffer for 1–1.5 h at RT. Finally, plates were washed, 3,3’,5,5’-Tetramethylbenzidine (TMB) (Thermo Fisher, Cat. 34029) was added, colour reaction was stopped with 20% sulfuric acid after 20 min of incubation and absorbance was measured at a wavelength of 450 nm using a BioTek SynergyHTX or a BioTek 800TS plate reader (Agilent BioTek). The ELISA endpoint titres were defined as the highest reciprocal serum or urine dilution that yielded a signal above the mean background signal plus 5-fold standard deviation (SD).

#### 2.12.2 Determination of functional antibody titres in sera by HAI assay

The HAI assay (33) was adapted to assess FimH inhibition by anti-FimH antibodies and antisera. Briefly, the UPEC strains UTI89, J96, CFT073 or EC958 were cultured at 37 °C under static conditions for 2 passages in Miller’s LB broth (BD Biosciences, Cat. 244610) to induce and enhance type 1 pilus (FimH) expression. The OD_600_ of the bacterial culture was determined and a suspension was generated with an OD_600_ of approximately 1.0 in PBS. 50 mL of this bacterial suspension were centrifuged at 3200 RCF for 20 min at 4 °C, the bacterial pellet was resuspended in 4.1 mL PBS and 300 μL of this final suspension was required for each HAI plate. Guinea pig RBCs were prepared by washing guinea pig blood in Alsever’s solution (Colorado Serum Company, Cat. 30100) in PBS and centrifugation at 1300 RCF for 6 min at 4 °C. RBCs were resuspended in PBS and diluted to a target OD640 of 1.95 ± 0.009. Bacterial and RBC suspensions were stored at 4 °C until use. The reaction mix was assembled in a sterile 96-well V-bottom plate in portrait orientation. A plate in which the mouse or rat sera were replaced by anti-FimH monoclonal antibody (mAb) 54G9 (EC_90_~1.6 μg/mL) served as positive control. This dilution strategy yielded a 2-dimensional matrix of serum/mAb concentration vs. bacterial concentration in the test wells. Sera/mAb were incubated with UPEC for 20 min at RT to allow for antibodies present in the serum to target and bind FimH expressed on UPEC. Finally, the prepared RBCs were added to each well and plates were incubated overnight at 4 °C. The resulting haemagglutination titre of the PBS control was defined as the number of wells (or bacterial dilutions) that showed haemagglutination, which was visually identified by a red hazy appearance as opposed to a discrete pellet of RBCs at the bottom of the V wells. The HAI effective concentration EC_90_ titre was defined as the reciprocal serum dilution that had a haemagglutination titre 16-fold (i.e., 4 wells) lower than the PBS control, which corresponds to >90% inhibition of haemagglutination.

#### 2.12.3 Determination of functional antibody titres in sera by BAI assay

The bacterial adhesion inhibition (BAI) assay is an alternative to the established HAI assay, measuring functional antibody responses against the homologous UPEC strain J96 by detecting antibody-mediated inhibition of *E. coli* binding to human bladder cells. Briefly, UPEC strain J96 was grown in LB broth for 2 days prior to use. After 2 days, bacteria were washed and labelled with fluorescein isothiocyanate (FITC). A cell count for the labelled bacteria was determined by measuring the OD_600_ of the suspension on a Nanodrop instrument and applying a 1 x 10^8^ cell number conversion factor. The labelled bacteria were adjusted to a concentration of 2.5 x 10^8^ bacterial cells/mL for final use in the assay. Serum samples in 1:2 dilutions were incubated for 1 h at 37 °C with the labelled bacteria to allow for antibodies present in the serum to target and bind FimH expressed on UPEC strain J96. The serum samples/bacteria mixtures were then added to plates containing human T24 bladder cells (BioAgilytix Labs) for 1 h at 37 °C to allow binding of bacteria to the bladder cells. T24 bladder cells were plated 1 day prior in a white 96-flat bottom plate at a density of 4 x 10^4^ cells/well in T24 growth medium (BioAgilytix Labs). Finally, plates were washed, and fluorescence signal was determined on a Synergy NEO2 plate reader (Agilent). As the bacteria were FITC-labelled, the presence of UPEC strain J96 in the final assay well was detectable via a fluorescent signal. Antibodies that prevent bacterial adherence to the T24 cells reduce the fluorescent signal in the assay. Sample data was normalised to a negative control tested in parallel on the assay plate that consisted of commercially available mouse or rat serum with an equivalent UPEC J96 dose as the samples. Dosed sample titrations results were plotted using a 4PL fit comparing normalised response to sample dilution. Response levels representing a 25% inhibition of signal (0.75 normalised response) and 50% inhibition of signal (0.50 normalised response) were interpolated from the 4PL curve of each dosed sample. Final BAI effective concentration EC_25_ or EC_50_ titres correspond to a reciprocal serum dilution resulting in 25% or 50% reduction, respectively, in UPEC binding to human uroepithelial cells.

#### 2.12.4 Detection of cellular immune responses in UPEC-naïve animals by ICS or ELISpot

FimH-specific T cell immune responses were analysed 2 weeks after the second or third immunisation with FimH_DG_-Ferritin mRNA by intracellular cytokine staining (ICS) in mice or enzyme linked immunospot (ELISpot) (R&D Systems, Cat. EL585) in rats. Isolated splenocytes were stimulated with a custom-made 15-mer overlapping peptide library spanning the full-length FimH. Splenocytes were thawed and seeded in 96-well round-bottom plates at a density of 2.5 x 10^6^ cells/mL for ICS (mouse) or in ELISpot plates (rat) containing α-MEM medium (Gibco, Cat. 22561-021) with 10% FCS, 100 U/mL penicillin, 100 mg/mL streptomycin, 2 mM L-glutamine and 10mM HEPES (Gibco, Cat. 15630-056). The cells were stimulated with the FimH peptide library at 1 μg/mL/peptide (mice splenocytes, in the presence of anti-CD28 (BD Biosciences, Cat. 553294, 1:400) and CD107α-PE-Cy7 (BioLegend, Cat. 121620, 1:100) or 5 μg/mL/peptide (rat splenocytes). Cells incubated with α-MEM medium containing DMSO (at a concentration corresponding to that of the peptide-stimulated samples) served as negative controls. For the ELISpot of rat splenocytes, cells stimulated with 5 ng/mL Phorbol 12-myristate 13-acetate and 500 ng/mL Ionomycin served as a positive control (data not shown). The ELISpot assay was performed according to the manufacturer’s instructions.

For mouse splenocyte ICS analysis, GolgiPlug (BD Biosciences, Cat. 555029,1:200) containing Brefeldin A was added after 1 h incubation at 37 °C, and the cells were incubated for additional 5–6 h at 37 °C. This was followed by a medium change to α-MEM medium and an incubation at 4 °C overnight. The following day, splenocytes were washed twice in PBS and incubated with LIVE/DEAD fixable Aqua Dye (Invitrogen, Cat. L34957,1:1000) at 4 °C for 30 min. After washing twice in PBS/0.5% BSA, the cells were stained with antibodies against CD90.2 (Thy1.2)-FITC (BioLegend, Cat. 140304, 1:200), CD8-APC-Cy7 (BioLegend, Cat. 100714, 1:200) and CD4-BD Horizon V450 (BD Biosciences, Cat.560468, 1:200) in the presence of Fcγ-blocking reagent (eBioscience, Cat. 14-0161-85, 1:100) for 30 min at 4 °C in PBS/0.5% BSA. Subsequently, cells were washed and fixed using Cytofix/Cytoperm (BD Biosciences, Cat. 554722) according to the manufacturer’s instructions. After washing twice in PermWash buffer (BD Biosciences, Cat. 554723), cells were incubated in PermWash buffer with anti-IFNγ-APC (BD Biosciences, Cat.554413, 1:100) and anti-TNF-PE (eBioscience, Cat.12-7321-82, 1:100) at 4 °C for 30 min. For flow cytometry analysis, splenocytes were washed twice in PermWash and finally resuspended in PBS with 0.5% BSA and 2 mM EDTA. Data was acquired on a ZE5 flow cytometer (Bio-Rad) and analysed using FlowJo software version 10.10.0.

#### 2.12.5 Quantification of cytokine and chemokine levels in the serum

Cytokines and chemokines were quantified in serum samples via a cytometric bead assay (CBA) using a LEGENDplex Multi-Analyte Flow Assay Kit, Mouse Anti-Virus Response Panel (13-plex) (BioLegend, Cat.740622) and LEGENDplex Rat Inflammation Panel (13-plex) (BioLegend, Cat.741396). The mouse panel measures CCL2, CCL5, CXCL1, CXCL10, GM-CSF, IFNα, IFNβ, IFNγ, IL-1β, IL-6, IL-10, IL-12p70, TNFα. The rat panel measures TNF, IL-10, IFNγ, CXCL1 (KC), CCL2 (MCP-1), GM-CSF, IL-18, IL-12p70, IL-1β, IL-17A, IL-33, IL-1α and IL-6.

Serum samples were diluted 1:2 (mice) or 1:4 (rat) and 12.5 μL of the dilutions were tested in single replicate. The beads were measured using a ZE5 flow cytometer (Bio-Rad) and analysed using the LEGENDplex data analysis software (version 2024-06-15).

For C3H/HeN mouse samples in the protective efficacy study, cytokine quantification was conducted using the DuoSet ELISA kits for CXCL1, IL-17, IL-6, and G-CSF in conjunction with the DuoSet Ancillary Reagent Kit 2 (R&D Systems, Cat. DY453, DY421, DY406, DY414, and DY008B). Bladder homogenate samples were thawed, centrifuged to remove particulates, diluted 1:10 (100 μL sample + 900 μL Reagent Diluent) and vortexed. Serum samples were diluted 1:10, 1:20 or 1:40 depending on remaining sample volume. Diluted samples (final volume 1 mL) were vortexed, aliquoted into 4 tubes (0.25 ml per tube), and frozen. A 7-point standard curve using 2-fold serial dilutions was prepared by diluting the provided standard with Reagent Diluent. ELISA plates from the DuoSet Ancillary Reagent Kit 2 were coated with 100 μL per well of 2 μg/mL of the appropriate capture antibody, and ELISA performed according to manufacturer’s instructions.

### 2.13 Statistical analyses

#### 2.13.1 Immunogenicity studies in UPEC-naïve animals

The sample size determination was informed by data and experience from previous mouse and rat studies (25), where the SD within a group for the log_10_-transformed binding antibody titre ranged between 0.35 and 0.83. To account for unknown variability in the current studies, a SD of 0.83 was assumed. Using this SD for the log_10_-transformed response data, 10 animals/group were required to detect a 10-fold (log_10_ = 1; corresponds to one sample titration step) difference between groups with 80% power in a one-sided t test (determined with G*Power version 3.1.9.4). Due to the natural variability in urine volume at sampling time points, 2 additional animals per group were included to ensure at least 10 samples per group were available for analysis. Consequently, 12 animals per vaccine group were used and all measurements were taken from distinct samples.

Statistical analysis between dosing schedules across all dose levels was performed using a two-way ANCOVA to assess differences. The study was adjusted for schedule and dose, including an interaction for schedule*dose. All binding and functional antibody data were log_X_-transformed for analysis, where X corresponded to the fold of the response titre. Testing of the null hypothesis (F statistic) that there is no difference between the effect of the 2-dose schedule and the 3-dose schedule on response, conditioned on dose, were provided (**Supplementary Data**). An ANCOVA without the interaction term was compared to check for collinearities. Before the ANCOVA analysis, the normality assumption was tested using the Shapiro-Wilk test and by examination of QQ plots. Due to low granularity of values (titres only reported at each fold), in some cases Shapiro-Wilk indicated possibly non-normal data, but the corresponding QQ plots demonstrated little to no bias, suggesting underlying normality. Correlations across all dose groups and both dose-schedules combined between serum HAI EC_90_ titres and serum BAI EC_25_ or EC_50_ titres were separately estimated using the Kendall’s Rank Correlation Coefficient.

These statistical analyses were all conducted in R (version 4.4.0 or higher, R Foundation for Statistical Computing) with RStudio as the graphic user interface. The ANCOVA was carried out using the aov() function from the *stats* package. The correlation analysis was carried out using the cor.test() function from the *stats* package. The corresponding *P* values and Kendall’s Rank Correlation Coefficients are represented in the results section or in the respective graphs. Differences were considered significant at *P* values <0.05.

#### 2.13.2 Protective efficacy study in UPEC-experienced animals

Sample size determination for the protective efficacy study followed the same assumptions as for the immunogenicity studies. To account for anticipated animal exclusions associated with UPEC sensitisation and persistent bacteriuria, 172 mice were enrolled, resulting in 147 mice included in the final challenge analysis (8–13 animals per treatment group). All measurements were taken from distinct samples.

For each dose schedule, statistically significant differences between the five treatment groups and the NaCl control group were assessed using an ordinary one-way ANOVA followed by Dunnett’s multiple comparison *post hoc* test for all challenge data, or a Kruskal-Wallis test followed by Dunn’s multiple comparison *post hoc* test for all binding and functional antibody data. All challenge data were log_10_-transformed for analysis. To test for normal or lognormal distribution, D’Agostino & Pearson, Anderson-Darling, Shapiro-Wilk, and Kolmogorov-Smirnov tests were used. These statistical analyses were all conducted using GraphPad Prism (version 10.4.1, GraphPad Software). Each *P* value was multiplicity adjusted to account for multiple comparisons. Differences were considered significant at adjusted *P* values of <0.05. Full statistical parameters and results are provided in **Supplementary Data**.

To analyse the relationship between functional antibodies in serum two weeks post last dose measured via the HAI assay and bacterial load in the bladder 24 h post challenge infection, a 3-step statistical correlation analysis was performed. Firstly, the relationship between HAI EC_90_ titres and bladder CFUs was analysed by using the linear model ln(CFUs) = α + β*ln(HAI) + ϵ, where ϵ is zero-mean Gaussian noise, and ln is the natural logarithm. Since no significant differences between 2-dose and 3-dose schedules was found, thereby rejecting the null hypothesis (dose effect 0.466, 95% CI (-0.577–1.508), *P* = 0.379), both datasets were combined for this 3-step correlation analysis. Secondly, the relationship between vaccine groups and HAI EC_90_ titres compared to both negative controls (datasets of NaCl control and irrelevant mRNA vaccine were combined as there were no significant differences between them) was analysed by using a one-way ANOVA with Dunnett’s multiple comparison *post hoc* test to check which treatments have mean HAI EC_90_ titre significantly different from negative control mean HAI EC_90_ titre. Finally, the relationship between vaccine groups, HAI EC_90_ titres and bladder CFUs was analysed using a one-way ANCOVA with HAI EC_90_ titres as a covariate with Dunnett’s multiple comparison *post hoc* test to examine the effect of vaccine choice on the bacterial load in the bladder. These statistical analyses were all conducted in R (version 4.4.0 or higher, R Foundation for Statistical Computing) with RStudio as the graphic user interface. The linear model was fit using the function lm() from the *stats* package; Dunnett’s test was done using the function DunnettTest() from the *DescTools* package. Full statistical parameters and results are provided in **Supplementary Data**.

## 3 Results

### 3.1 The mRNA-encoded FimH_DG_-Ferritin fusion protein assembles into PNPs with intact, functional surface FimH epitopes *in vitro*

**Figure 1:**
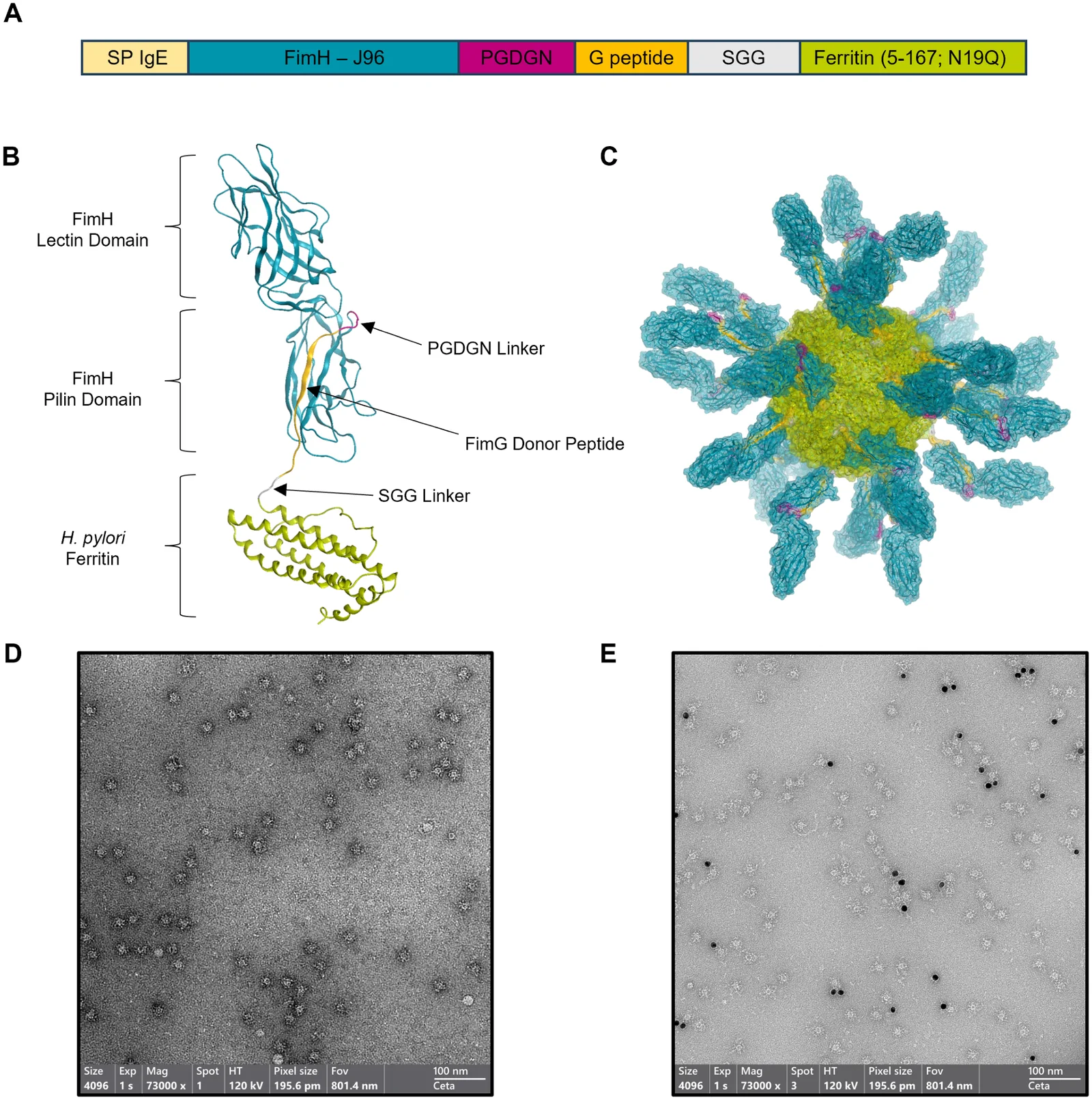
FimH_DG_-Ferritin mRNA encodes FimH_DG_-Ferritin PNPs that are assembled with intact, functional, surface FimH epitopes *in vitro.* Schematic representation of the **(A)** FimH_DG_-Ferritin construct, **(B)** structural models of the monomer, and **(C)** assembled PNP. FimH_DG_-Ferritin contains an N-terminal signal peptide derived from human IgE (SP IgE, yellow) facilitating the secretion of the protein in mammalian cells. The FimH sequence, derived from UPEC strain J96 (cyan), is divided into the lectin domain, containing the mannose-binding site, and pilin domain, which interacts with FimG. The pilin domain is fused to a sequence from FimG (G peptide, gold), completing the Ig-fold of the pilin domain. The FimG peptide is attached to FimH via a PGDGN linker (pink) and fused to *H. pylori* ferritin (light green) via a SGG linker (grey). The *H. pylori* ferritin contains a mutation (N19Q), preventing glycosylation at aa position N19 and a deletion of aa 1−4. **(D, E)** Negative stain EM of purified FimH_DG_-Ferritin **(E)** with FimH-specific immunogold labelling. The structural model was generated using CCG MOE software. aa, amino acid; CCG, Chemical Computing Group; EM, electron microscopy; *H. pylori*, *Helicobacter pylori*; MOE, Molecular Operating Environment; mRNA, messenger RNA; PGDGN, proline-glycine-aspartic acid-glycine-asparagine; PNP, protein nanoparticle; SGG, serine-glycine-glycine; SP, signal peptide; UPEC, uropathogenic *E. coli.*.

The development of the FimH_DG_-Ferritin mRNA construct has been described previously (25). Briefly, a 5 aa linker (PGDGN) connects UPEC strain J96-derived FimH and the FimG donor peptide (FimH_DG_); this complex is fused to *H. pylori* ferritin via a short SGG linker (**Figures 1A, B**) and predicted to self-assemble into a 24-mer FimH_DG_-Ferritin PNP, with FimH_DG_ displayed on the surface (**Figure 1C**).

To confirm the assembly of PNPs *in vitro*, FimH_DG_-Ferritin mRNA constructs were introduced into HeLa cells through mRNA-transfection. PNP production was confirmed in the supernatant by nPAGE and western blot for FimH following mannose-agarose affinity purification, where signals at high molecular weights (above 1048 kDa) indicated the presence of PNPs (**Supplementary Figure 1A**). To aid purification, a His-tagged version of FimH_DG_-Ferritin was produced. nPAGE analysis confirmed the PNP formation (**Supplementary Figure 1B**) and verified that the His-tag had no impact on the oligomeric assembly state (**Supplementary Figure 1C**). Variations in protein size observed between cell lysate- and supernatant-extracted monomeric fusion proteins were assessed via PNGase F treatment, indicating that lysates contained incompletely glycosylated protein, whereas most secreted FimH_DG_-Ferritin fusion proteins were fully glycosylated (**Supplementary Figure 2**).

Negative stain EM of purified, secreted His-tagged FimH_DG_-Ferritin PNPs revealed a homogeneous population of star-shaped particles, indicating presence of FimH_DG_ on the ferritin scaffold (**Figure 1D**). The particles measured 20–30 nm in diameter, aligning with the anticipated size of ~29 nm. Incubation of purified PNPs with gold-labelled monoclonal antibody, which targets a functional epitope of FimH and thereby inhibits mannose binding, resulted in gold particle attachment to the outer protrusions of PNPs, demonstrating specific antibody-FimH interaction (**Figure 1E**). Together, these data confirmed that FimH_DG_-Ferritin mRNA transfection *in vitro* results in the formation of PNPs displaying correctly folded, surface FimH proteins with intact functional epitopes.

### 3.2 The FimH_DG_-Ferritin mRNA vaccine induces dose-dependent binding antibody responses in serum and urine of UPEC-naïve animals

**Figure 2:**
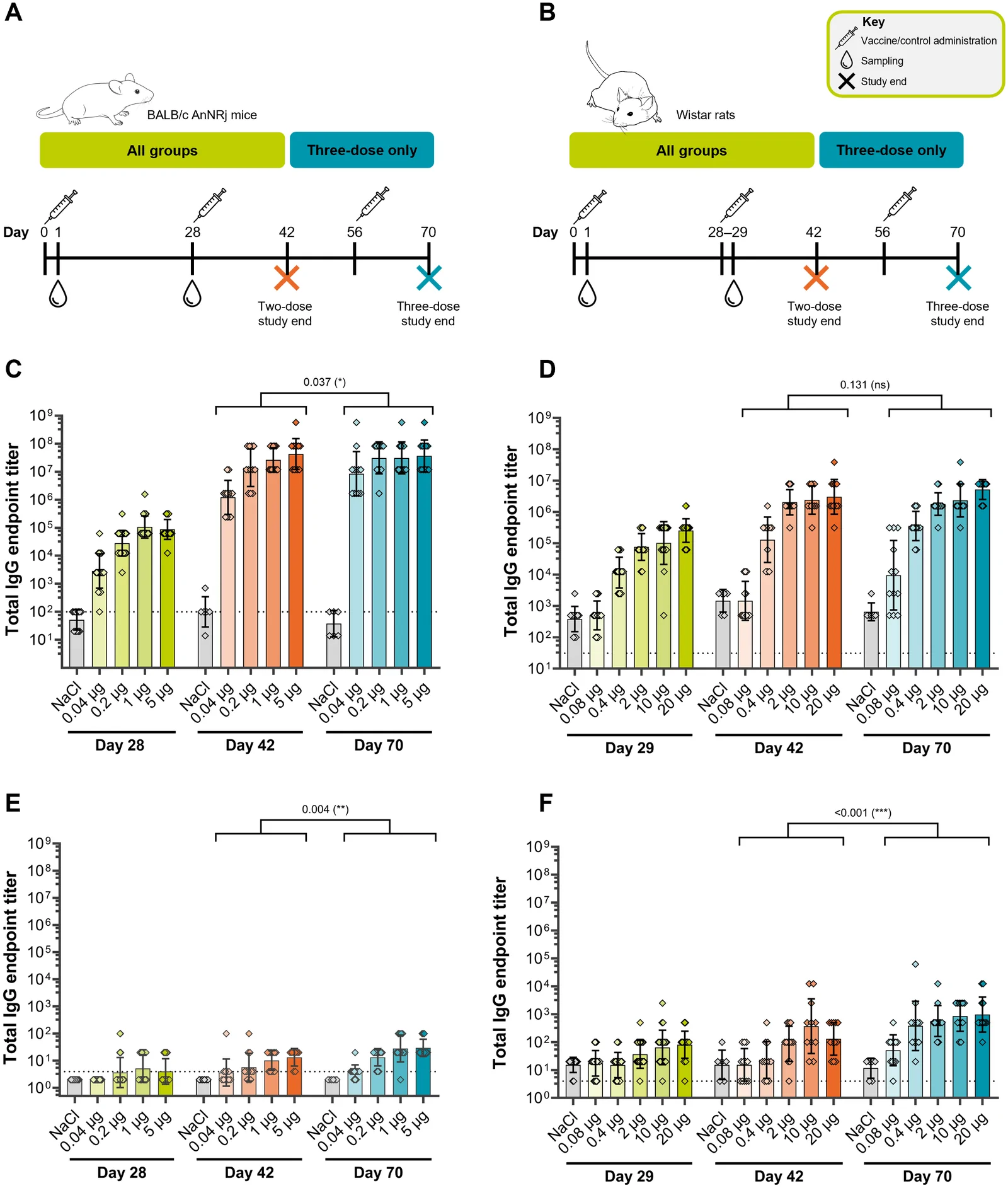
FimH_DG_-Ferritin mRNA vaccination induces dose-dependent binding antibody responses in serum and urine of UPEC-naïve animals. FimH-specific IgG binding antibodies were measured by ELISA in serum and urine of UPEC-naïve animals in dose-response immunogenicity studies in **(A, C, E)** female BALB/c AnNRj mice (n=12/dose schedule) and **(B, D, F)** female Wistar rats (n=12/dose schedule). Animals received either 2 or 3 IM administrations of the FimH_DG_-Ferritin mRNA vaccine on Days 0, 28 (all) and 56 (3-dose only) at doses of 0.04, 0.2, 1 or 5 μg (mice) and 0.08, 0.4, 2, 10 or 20 μg (rats). Control animals received 0.9% NaCl (n=6/dose schedule). Anti-FimH IgG endpoint titres were measured in **(C, D)** serum or **(E, F)** urine collected 4 weeks post first administration (Day 28/29, green bars, 2- and 3-dose schedule), 2 weeks post second administration (Day 42, orange bars, 2-dose schedule only) and 2 weeks post third administration (Day 70, blue bars). Due to limited sample availability, sample sizes for specific groups were reduced as follows: **(D)** Day 29: 0.4 µg (n=23), 10 µg (n=23); Day 42: 0.4 µg (n=11), 10 µg (n=11); **(E)** Day 28: NaCl (n=11), 5 µg (n=23); Day 42: 0.04 µg (n=11), 5 µg (n=8); **(F)** Day 29: 0.4 µg (n=23), 10 µg (n=22), 20 µg (n=23); Day 42: 0.4 µg (n=11), 10 µg (n=11). Each dot represents an individual animal, bars depict the geometric mean, error bars depict the geometric SD. Dotted lines represent the respective LLOQ. The statistical difference between 2- and 3-dose schedules across all dose levels was analysed using a two-way ANCOVA (*P < 0.05, **P < 0.01, ***P < 0.001; ns, not significant). ANCOVA, Analysis of Covariance; ELISA, enzyme-linked immunosorbent assay; IM, intramuscular; LLOQ, lower limit of quantification; mRNA, messenger RNA; SD, standard deviation; UPEC, uropathogenic *E. coli.*.

The immunogenicity of the FimH_DG_-Ferritin mRNA vaccine was assessed in female BALB/c AnNRj mice and Wistar rats. UPEC-naïve animals received either 2 or 3 IM administrations of FimH_DG_-Ferritin mRNA vaccine at doses of 0.04, 0.2, 1 or 5 μg in mice and 0.08, 0.4, 2,10 or 20 μg in rats, or 0.9% NaCl as a negative control, with doses administered 4 weeks apart (**Figures 2A, B**). Early innate immune responses were assessed by quantification of serum cytokines and chemokines 18 h after the first administration of the FimH_DG_-Ferritin mRNA vaccine (**Supplementary Figure 3**). In mice, IFNα, IL-6, IFNγ, CXCL1, CCL2, and CXCL10 were induced in a dose-dependent manner, with highest levels observed at the 5 μg dose (**Supplementary Figures 3A–F**). In rats, induction of IL-6 and CCL2 was confirmed, accompanied by a dose-dependent decrease in IL-10 (**Supplementary Figures 3G–I**). All other measured cytokines and chemokines remained comparable to the negative control (data not shown).

The induction of anti-FimH and anti-ferritin IgG binding antibodies was assessed by ELISA 4 weeks after the first administration and 2 weeks after the second and/or third administration. In mice, all FimH_DG_-Ferritin mRNA vaccine doses elicited a dose-dependent increase in FimH-specific serum IgG compared with the negative control (**Figure 2C**). The second administration significantly increased mouse serum anti-FimH IgG binding antibodies across all dose levels; antibody titres reached a plateau at ≥0.2 μg after 3 administrations. In rats, serum binding antibodies also increased in a dose-dependent manner from 0.4 to 20 μg after the first and second administration, but only the lower dose groups showed an additional boost after the third administration (**Figure 2D**). The FimH_DG_-Ferritin mRNA vaccine also induced a dose-dependent increase in serum anti-*H. pylori* ferritin antibodies in both species (**Supplementary Figures 4A, B**). The potential for cross-reactivity of induced IgG antibodies against human ferritin light (**Supplementary Figures 4C, D**) and heavy chain (**Supplementary Figures 4E, F**) was assessed, with titres at or slightly above the lower limit of quantification (LLOQ) after 2 and 3 administrations across all doses, similar to the negative control.

In mouse urine, anti-FimH binding antibodies were detected at low levels after the first FimH_DG_-Ferritin mRNA vaccine administration. A clear dose-dependent increase was observed following the second and third administration for doses above 0.2 μg (**Figure 2E**). In rats, urinary anti-FimH binding antibodies were detectable after 1 or 2 doses at ≥2 μg (**Figure 2F**). In both species, the third administration consistently enhanced urinary anti-FimH IgG binding antibody levels across all dose groups (**Figures 2E, F**).

### 3.3 The FimH_DG_-Ferritin mRNA vaccine induces dose-dependent functional antibody responses against homologous and heterologous UPEC strains in serum of UPEC-naïve animals

Functional antibody responses, defined as FimH-specific antibodies capable of preventing UPEC binding to target cells in a mannose-dependent manner, were assessed at Day 42 (2-dose schedule) and Day 70 (3-dose schedule). Sera from mice and rats were analysed by (i) haemagglutination inhibition (HAI) assay, which measures FimH-dependent agglutination of guinea pig RBCs and (ii) BAI assay, which quantifies antibody-mediated inhibition of *E. coli* binding to human bladder epithelial cells.

**Figure 3:**
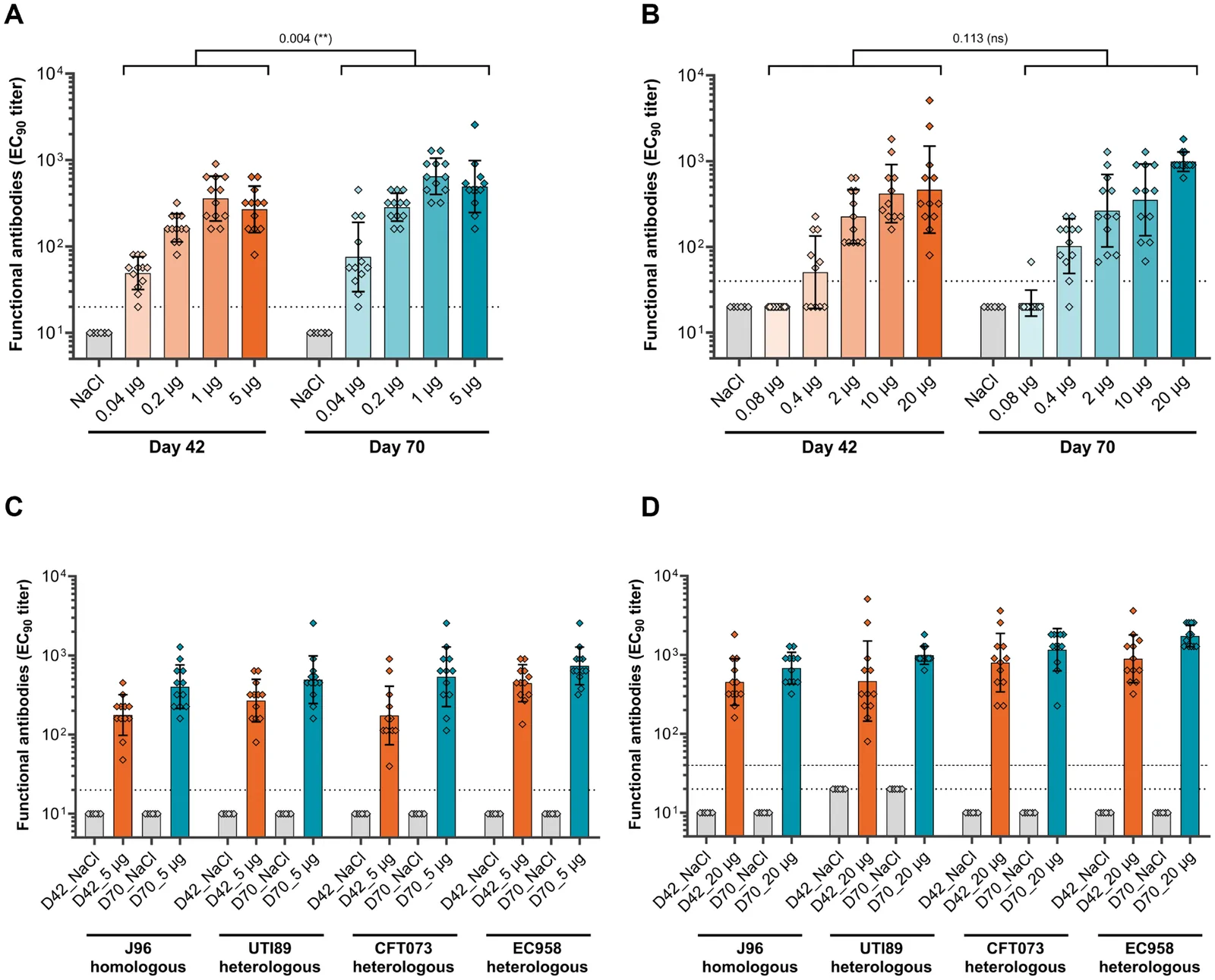
FimH_DG_-Ferritin mRNA vaccination induces dose-dependent functional antibody responses against homologous and heterologous UPEC strains in serum of UPEC-naïve animals. FimH-specific functional antibodies were measured by HAI assay in serum of UPEC-naïve animals in dose-response immunogenicity studies in **(A, C)** female BALB/c AnNRj mice (n=12/dose schedule) and **(B, D)** female Wistar rats (n=12/dose schedule). Animals received either 2 or 3 IM administrations of FimH_DG_-Ferritin mRNA vaccine on Days 0, 28 (all) and 56 (3-dose only) at doses of 0.04, 0.2, 1 or 5 μg (mice) and 0.08, 0.4, 2, 10 or 20 μg (rats). Control animals received 0.9% NaCl (n=6/dose schedule). HAI EC_90_ titres against **(A–D)** the heterologous UPEC strain UTI89^†^ and **(C, D)** the homologous UPEC strain J96 and heterologous strains CFT073 and EC958 were measured in serum collected two weeks post second administration (Day 42, orange bars, 2-dose schedule only) or two weeks post third administration (Day 70, blue bars). HAI titres are reported as EC_90_ values indicating the reciprocal serum dilution resulting in >90% inhibition of haemagglutination. Due to limited sample availability, sample sizes for specific groups were reduced as follows: **(B)** Day 42: 0.4 µg (n=11), 10 µg (n=11). Each dot represents an individual animal, bars depict the geometric mean, error bars depict the geometric SD. Dotted lines represent the respective LLOQ; the dashed line in **(D)** represents the LLOQ for UPEC strain UTI89. Values below the LLOQ were set to half of the respective LLOQ. The statistical difference between 2- and 3-dose schedules across all dose levels in **(A)** and **(B)** was analysed using a two-way ANCOVA (ns: not significant). ^†^Same data in panel **(A)** and **(C)** as well as in panel **(B)** and **(D)**, respectively. ANCOVA, Analysis of Covariance; HAI, haemagglutination inhibition; IM, intramuscular; LLOQ, lower limit of quantification; mRNA, messenger RNA; SD, standard deviation; UPEC, uropathogenic *E. coli.*.

Functional antibodies against the heterologous UPEC strain UTI89 were quantified by HAI assay (**Figures 3A, B**). Both dose schedules induced a dose-dependent increase in HAI EC_90_ titres starting at 0.04 μg in mice, and 0.4 μg in rats, with the exception of a modest decrease at the 5 μg dose relative to the 1 μg dose in mice. Relative to two administrations, three administrations significantly improved HAI EC_90_ titres in mice, whereas a third administration in rats yielded no statistically significant increase across all doses.

To examine cross-strain functionality, sera from animals that exhibited high UPEC UTI89-specific HAI EC_90_ titres (5 µg in mice; 20 µg in rats), were tested against additional UPEC strains: 1) the homologous J96 strain (34), isolated from a pyelonephritis case; 2) the heterologous CFT073 strain (35), isolated from an acute pyelonephritis case; and 3) EC958 (36), a heterologous extended spectrum beta-lactamase (ESBL)-producing, sequence-type 131 (ST131) strain, resistant to several antibiotics. These strains were selected for their heterogeneous *fimH* sequence types and FimH protein variants (**Supplementary Table 1**). For comparison, UTI89-specific HAI EC_90_ titres from the same dose groups (**Figures 3A, B**) are presented alongside the additional tested strains (**Figures 3C, D**). In both mice and rats, functional antibody titres were detected against all tested UPEC strains following 2 or 3 administrations of the FimH_DG_-Ferritin mRNA vaccine (**Figures 3C, D**). Heterologous responses were equivalent to or slightly higher than titres against the homologous J96 strain.

BAI assay analysis of immune sera from mice and rats confirmed that the FimH_DG_-Ferritin mRNA vaccine induced functional antibodies that inhibit the binding of homologous UPEC strain J96 to bladder epithelial cells, with no significant difference between the 2- or 3-dose schedules (**Supplementary Figure 5**). Across all dose groups and both dose-schedules combined, correlation analyses between heterologous UTI89 HAI EC_90_ titres (**Figures 3A, B**) and homologous J96 BAI titres (**Supplementary Figure 5**) confirmed a strong, statistically significant positive correlation between HAI EC_90_ and both BAI EC_25_ and EC_50_ titres in both species. Kendall’s Rank Correlation Coefficients were τ = 0.559 (BAI EC_25_) and τ = 0.561 (BAI EC_50_) in mice, and τ = 0.573 (BAI EC_25_) and τ = 0.597 (BAI EC_50_) in rats (all *P* < 0.001).

### 3.4 The FimH_DG_-Ferritin mRNA vaccine induces FimH-specific cellular immune responses in UPEC-naïve animals

**Figure 4:**
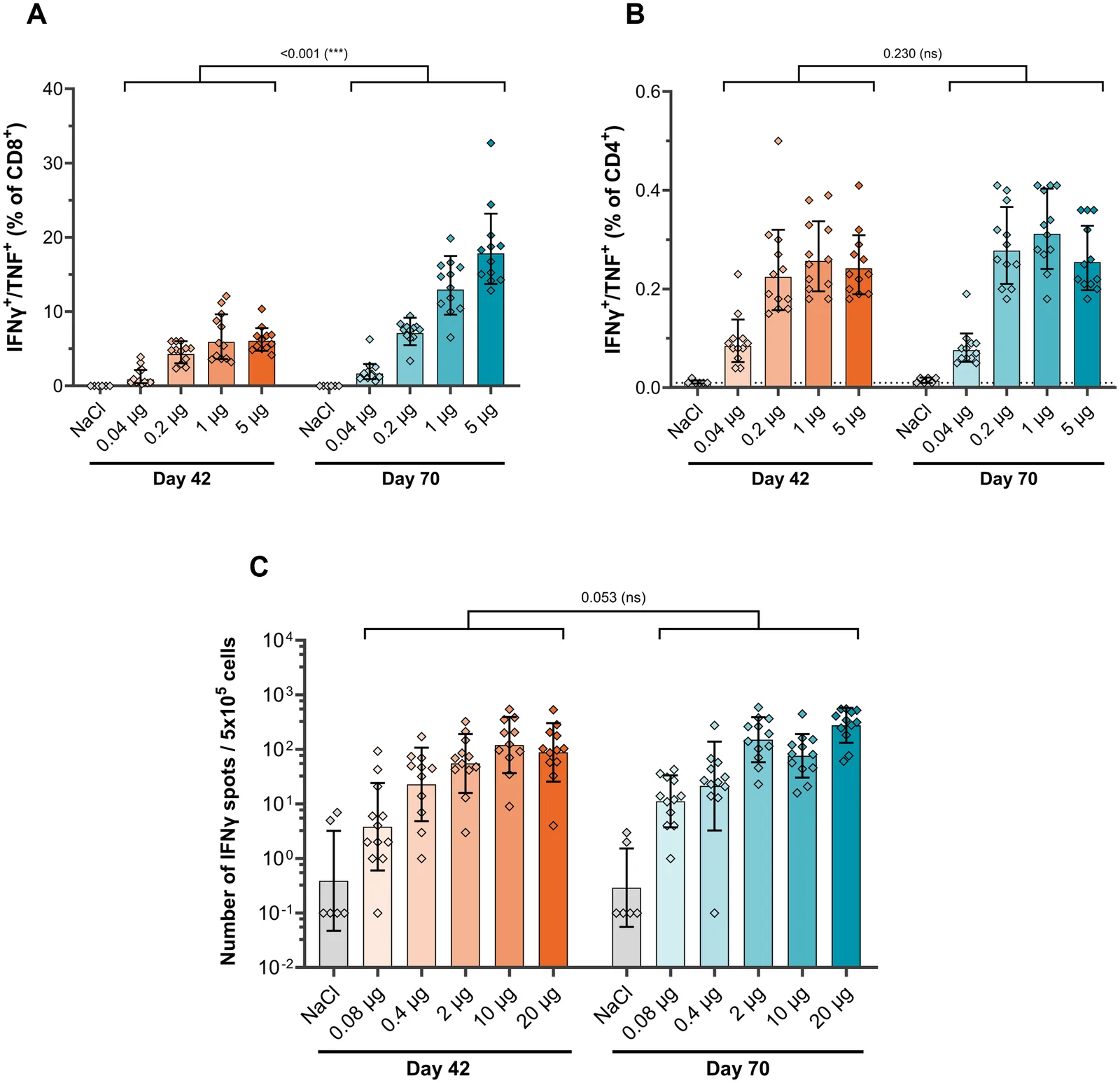
FimH_DG_-Ferritin mRNA vaccination induces FimH-specific cellular immune responses in the spleen of UPEC-naïve animals. FimH-specific T cell responses were measured in the splenocytes of FimH_DG_-Ferritin mRNA-vaccinated animals in dose-response immunogenicity studies in **(A, B)** female BALB/c AnNRj mice (n=12/dose schedule) and **(C)** female Wistar rats (n=12/dose schedule). UPEC-naïve animals received either 2 or 3 IM administrations of FimH_DG_-Ferritin mRNA vaccine on Days 0, 28 (all) and 56 (3-dose only) at doses of 0.04, 0.2, 1 or 5 μg (mice) and 0.08, 0.4, 2, 10 or 20 μg (rats). Control animals received 0.9% NaCl (n=6/dose schedule). Isolated splenocytes were re-stimulated with a 15-mer peptide library spanning the FimH full length protein. FimH-specific **(A)** CD8^+^ and **(B)** CD4^+^ and **(C)** IFNγ-secreting T cell responses were analysed in **(A, B)** mice samples by ICS and flow cytometry (gating analysis shown in **Supplementary Figure 6**) and **(C)** rat samples by ELISpot, two weeks post second administration (Day 42, orange bars, 2-dose schedule only) or third administration (Day 70, blue bars). Due to limited sample availability, sample sizes for specific groups were reduced as follows: **(C)** Day 42: 0.4 µg (n=11), 10 µg (n=11). Each dot represents an individual animal, bars depict the geometric mean, error bars depict the geometric SD. **(A, B)** Dotted lines represent the respective LLOQ. The statistical difference between 2- and 3-dose schedules across all dose levels was analysed using a two-way ANCOVA (***P < 0.001; ns: not significant). ANCOVA, Analysis of Covariance; CD, cluster of differentiation; ELISpot, enzyme linked immunospot; ICS, intracellular cytokine staining; IM, intramuscular; LLOQ, lower limit of quantification; mRNA, messenger RNA; SD, standard deviation TNF, tumour necrosis factor; UPEC, uropathogenic *E. coli.*.

FimH-specific cellular responses were assessed in splenocytes collected from mice and rats isolated 2 weeks after the second or third administrations. Splenocytes were stimulated with a FimH peptide library and T cell responses were quantified by ICS and flow cytometry in mice (see **Supplementary Figure 6** for gating strategy) or ELISpot in rats. In mice, both dosing schedules induced dose-dependent increases in IFNγ^+^TNF^+^ FimH-specific CD4^+^ and CD8^+^ T cells, with CD8^+^ T cell responses significantly enhanced after the third dose across all dose levels (**Figures 4A, B**). In rats, IFNγ secreting FimH-specific T cells were induced following 2 or 3 vaccine administrations (**Figure 4C**).

### 3.5 The FimH_DG_-Ferritin mRNA vaccine demonstrates protective efficacy against UPEC infection in a rUTI mouse model

**Figure 5:**
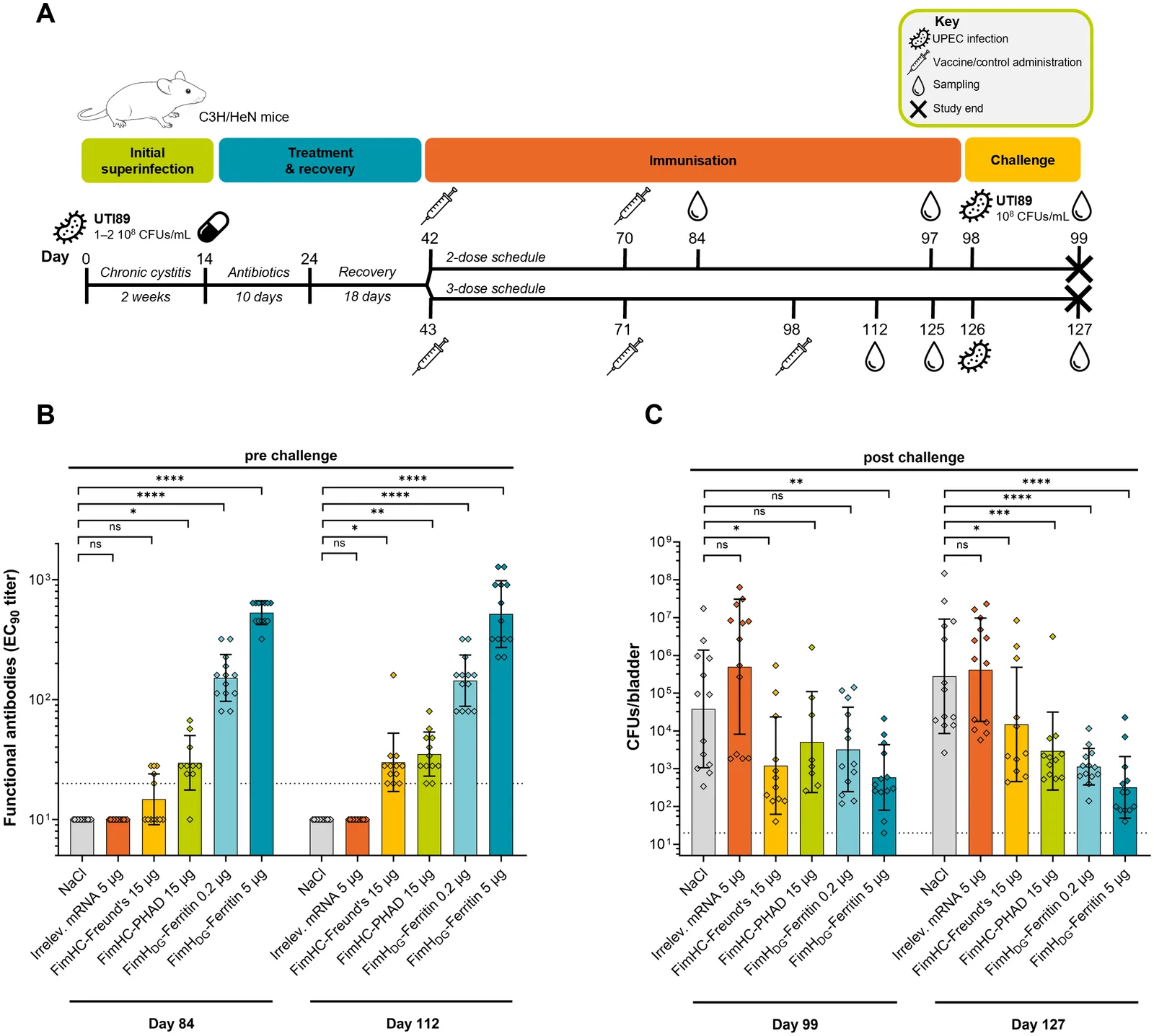
FimH_DG_-Ferritin mRNA vaccination demonstrates protective efficacy against UPECinfection in the bladder of an rUTI mouse model. **(A)** Protective efficacy study schematic evaluating FimH_DG_-Ferritin mRNA vaccine in the rUTI mouse model. C3H/HeN mice were sensitised by superinfection with UPEC strain UTI89 to develop chronic cystitis, which was subsequently treated with antibiotics. On Day 42 (2-dose schedule) or Day 43 (3-dose schedule), sensitised mice were injected IM with either 0.2 or 5.0 μg/dose of the FimH_DG_-Ferritin mRNA vaccine, with 0.9% NaCl solution and irrelevant mRNA vaccine as negative controls, and FimHC protein complex vaccination (adjuvanted with either Freund’s adjuvant or PHAD) as vaccine controls. Vaccine doses were given 4 weeks apart and challenge infections occurred 4 weeks after the final vaccine dose. Mice were sacrificed 24 h post challenge infection. **(B)** FimH-specific functional antibody responses against the heterologous UPEC strain UTI89 were measured by HAI assay in serum collected two weeks prior to challenge in the 2-dose schedule (Day 84) and 3-dose schedule (Day 112). HAI titres are reported as EC_90_ values indicating the reciprocal serum dilution resulting in >90% inhibition of haemagglutination. Sample sizes (n) for the 2-dose schedule were: NaCl, 13; irrelevant mRNA, 13; FimHC-Freund’s, 12; FimHC-PHAD, 10; FimH_DG_-Ferritin 0.2 µg, 13; FimH_DG_-Ferritin 5 µg, 13. For the 3-dose schedule, n values were: NaCl, 13; irrelevant mRNA, 13; FimHC-Freund’s, 12; FimHC-PHAD, 12; FimH_DG_-Ferritin 0.2 µg, 13; FimH_DG_-Ferritin 5 µg, 13. **(C)** Bladder bacterial burden determined 24 h post challenge infection in sensitised mice from 2-dose schedule (Day 99) or 3-dose schedule (Day 127). Sample sizes (n) for the 2-dose schedule were: NaCl, 13; irrelevant mRNA, 13; FimHC-Freund’s, 12; FimHC-PHAD, 8; FimH_DG_-Ferritin 0.2 µg, 13; FimH_DG_-Ferritin 5 µg, 13. For the 3-dose schedule, n values were: NaCl, 13; irrelevant mRNA, 13; FimHC-Freund’s, 11; FimHC-PHAD, 12; FimH_DG_-Ferritin 0.2 µg, 13; FimH_DG_-Ferritin 5 µg, 13. **(B, C)** Each dot represents an individual animal, bars depict the geometric mean, error bars depict the geometric SD. Dotted lines represent the respective LLOQ. Significant differences between the treatment groups and the NaCl control group were determined by **(B)** Kruskal-Wallis test with Dunn´s multiple comparison *post hoc* test or **(C)** one-way ANOVA with Dunnett’s multiple comparison *post hoc* test of log_10_-transformed data, (*P < 0.05; **P < 0.01; ***P < 0.001; ****P < 0.0001; ns, not significant). ANOVA, Analysis of Variance; CFUs, colony forming units; h, hours; HAI, haemagglutination inhibition; IM, intramuscular; LLOQ, lower limit of quantification; mRNA, messenger RNA; PHAD, Phosphorylated HexaAcyl Disaccharide; rUTI; recurrent urinary tract infection; SD, standard deviation; UPEC, uropathogenic *E. coli*.

Finally, the protective efficacy of the FimH_DG_-Ferritin mRNA vaccine-induced anti-FimH adaptive immune response was evaluated in a recurrent cystitis model in C3H/HeN mice following UPEC challenge (37–39) (**Figure 5A**). To sensitise mice to rUTI, chronic cystitis was established through an initial superinfection delivered via transurethral inoculation of UPEC strain UTI89 (29), followed by antibiotic treatment initiated on Day 14, and an 18-day convalescence. Mice were immunised intramuscularly on Day 42 with either 0.2 or 5.0 µg/dose of the FimH_DG_-Ferritin mRNA vaccine, followed by 1 or 2 additional doses on Days 70 and 98, respectively. Negative controls consisted of mice receiving either 0.9% NaCl solution or an irrelevant mRNA-LNP vaccine encoding the rabies virus glycoprotein, to account for non-specific mRNA-LNP effects. Vaccine control mice were immunised with 15 µg FimHC (also designated “FimCH”) protein complex, adjuvanted with either Freund’s adjuvant (FimHC-Freund’s) or Phosphorylated HexaAcyl Disaccharide (PHAD) (FimHC-PHAD). All doses were administered 4 weeks apart, with the final immunisation 4 weeks before challenge.

Pre-challenge functional and binding serum antibody responses were quantified by HAI assay and ELISA, respectively. Both doses of the FimH_DG_-Ferritin mRNA vaccine induced significantly higher HAI EC_90_ titres against the heterologous UPEC strain UTI89 than the NaCl control (**Figure 5B**). Notably, the magnitude of vaccine-induced HAI EC_90_ titres in UPEC-experienced C3H/HeN mice was broadly comparable to that observed in UPEC-naïve BALB/c mice receiving the same vaccine doses and immunisation schedules (**Figure 3A**), with geometric mean titres generally differing by no more than a single assay dilution step (two-fold) across the corresponding dose groups and immunisation schedules. In contrast to the UPEC-naïve BALB/c mouse model, comparable functional antibody levels were observed after either 2 or 3 immunisations in the C3H/HeN efficacy model (**Figure 5B**). A similar pattern was observed with FimHC-PHAD, where a third dose did not further increase functional titres. Conversely, mice immunised with FimHC-Freund’s showed a clear increase in functional antibody titre from a third dose. All FimH vaccines elicited significantly elevated FimH-specific serum IgG binding antibodies compared with NaCl control (**Supplementary Figure 7**).

To evaluate vaccine-mediated protection against UTI infection, immunised, sensitised mice were challenged with UPEC strain UTI89. Bacterial burdens in bladder tissue 24 h post-challenge served as the primary endpoint (**Figure 5C**). A statistical analysis found no significant differences between animals receiving NaCl vs irrelevant mRNA-LNP and between vaccinated animals in 2 vs 3-dose schedules (**Supplementary Data**). Pooling these data revealed that, relative to the combined negative control groups, immunisation with FimHC-Freund’s or FimHC-PHAD reduced bladder CFUs 54.1-fold (95% CI: 349, 8.4) and 59.6-fold (95% CI: 422, 8.4), respectively, whereas the FimH_DG_-Ferritin mRNA vaccine achieved greater reductions of 114-fold (0.2 µg dose; 95% CI: 678, 19) and 501-fold (5 µg dose; 95% CI: 2996, 84). Thus, mice receiving the FimH_DG_-Ferritin mRNA vaccine exhibited the most pronounced decrease in bacterial attachment to and/or invasion of bladder tissue. A correlation analysis revealed a significant relationship between pre-challenge serum functional antibody levels (HAI EC_90_ titres; **Figure 5B**) and bladder bacterial load 24 h post infection (**Figure 5C**): each 10-fold increase in HAI titre corresponded to an estimated 22.4-fold (95% CI: 48.5, 10.3) reduction in bladder bacterial burden (**Supplementary Data**).

**Figure 6:**
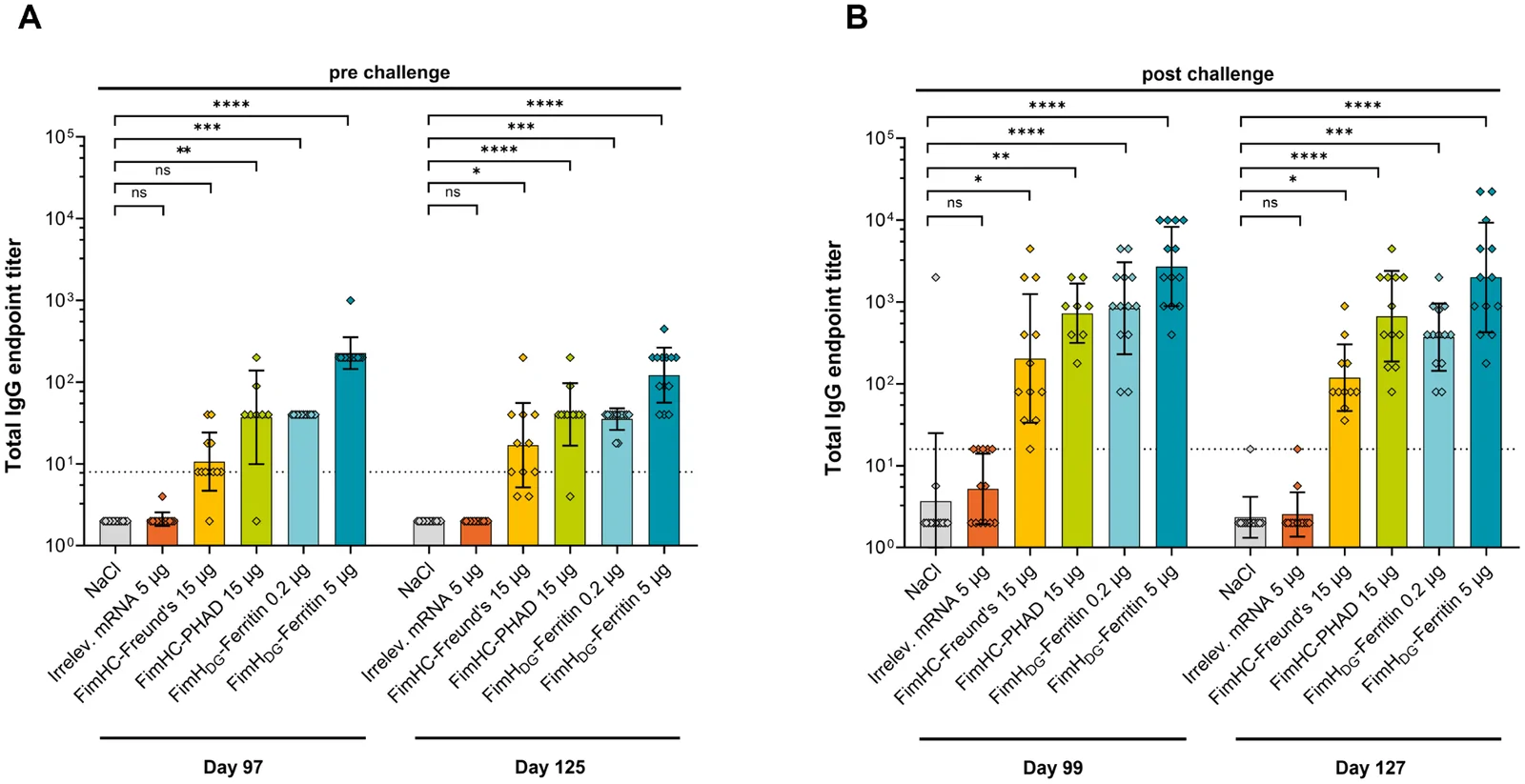
FimH_DG_-Ferritin mRNA vaccination induces anti-FimH IgG responses in urine that may confer protection against UPEC in a rUTI mouse model. FimH-specific IgG binding antibodies in urine were measured by ELISA in the rUTI mouse model using C3H/HeN mice. Urine was collected **(A)** 24 h pre-challenge infection (Day 97 and 125 in 2-dose and 3-dose schedule mice, respectively) and **(B)** 24 h post-challenge (Day 99 and 127 in 2-dose and 3-dose schedule mice, respectively). **(A, B)** Sample sizes (n) for the 2-dose schedule were: NaCl, 13; irrelevant mRNA, 13; FimHC-Freund’s, 12; FimHC-PHAD, 8; FimH_DG_-Ferritin 0.2 µg, 13; FimH_DG_-Ferritin 5 µg, 13. For the 3-dose schedule, n values were: NaCl, 13; irrelevant mRNA, 13; FimHC-Freund’s, 11; FimHC-PHAD, 12; FimH_DG_-Ferritin 0.2 µg, 13; FimH_DG_-Ferritin 5 µg, 13. Each dot represents an individual animal (geometric mean of two replicates), bars depict the geometric mean, error bars depict the geometric SD. Dotted lines represent the respective LLOQ. Values below the LLOQ (8 in **(A)**, 16 in **(B)**) were set to 2 for each individual replicate. Statistically significant differences between the treatment groups and the NaCl control group were determined by Kruskal-Wallis test with Dunn’s multiple comparison *post hoc* test (*P < 0.05; **P < 0.01; ***P < 0.001; ****P < 0.0001; ns, not significant). ELISA, enzyme-linked immunosorbent assay; h, hours; LLOQ, lower limit of quantification; mRNA, messenger RNA; PHAD, Phosphorylated HexaAcyl Disaccharide; rUTI, recurrent urinary tract infection; SD, standard deviation; UPEC, uropathogenic *E. coli*.

Urine samples collected 24 h pre- and post-challenge were analysed for anti-FimH IgG binding antibodies by ELISA (**Figure 6**). FimHC protein complex and FimH_DG_-Ferritin mRNA vaccination induced increased levels of both pre- and post-challenge anti-FimH binding antibodies, relative to NaCl control (**Figures 6A, B**). Across all vaccine groups and dose schedules, urinary anti-FimH IgG titres were, on average, 15-fold higher 24 h post-challenge compared with 24 h pre-challenge levels. In all readouts, there was no significant difference between the irrelevant mRNA vaccine and NaCl control (**Figures 5B, C**, **6A, B**, **Supplementary Figures 7**, **8**). To further explore protective mechanisms in the bladder, selected innate immune factors associated with UTI pathogenesis were examined 24 h post-challenge. FimH_DG_-mRNA vaccination did not significantly impact IL-17 or IL-6 relative to the NaCl control (data not shown); however, significantly lower levels of CXCL1 were measured in the bladder and serum, along with reduced serum G-CSF (**Supplementary Figure 8**), indicating reduced inflammation and correspondingly milder disease outcome following bacterial challenge.

## 4 Discussion

Our findings demonstrate that the FimH_DG_-Ferritin mRNA vaccine confers protective efficacy in a rUTI animal model through the generation of immune-accessible FimH_DG_-Ferritin PNPs that elicit strong, cross-reactive immune responses against multiple UPEC strains. To our knowledge, this is the first human-intended UPEC vaccine to show protective efficacy in this setting.

Demonstrating *in vivo* protection in an animal model is a critical milestone for vaccine development and supports advancement towards clinical testing. We found that FimH_DG_-Ferritin mRNA vaccination induced a dose-dependent adaptive immune response that significantly protected UPEC-experienced mice from challenge infection and reduced severe disease compared with controls. The rUTI mouse model recapitulates key aspects of human UTI susceptibility, disease pathogenesis, and bladder tissue remodelling (37–39), thereby providing a biologically relevant challenge model. Importantly, this model also reflects a clinically relevant setting in which vaccination is administered to hosts with prior UPEC exposure and a history of infection, rather than to immunologically naïve individuals. Consequently, this model may better reflect the immunological and pathological environment of the population most likely to receive a UPEC vaccine. To achieve consistent infection, 10^8^ CFUs/mouse is required, which exceeds the bacterial burden typically observed in human diagnostic cultures (~10^5^ CFUs/mL) (40) but is consistent with other animal models, including non-human primates (41). A high bacterial inoculum may reduce sensitivity for detecting partial protection, as neutralised bacteria can contribute to background signal and reduce dynamic range (38). Although this represents a limitation of the study, it also establishes a stringent challenge model. The ability of the FimH_DG_-Ferritin mRNA vaccine to confer protective efficacy in this model is therefore especially remarkable and underscores the significance of our findings. Relative to FimHC-Freund’s and FimHC-PHAD vaccine controls, formulated to match the only FimH-targeting vaccines with efficacy *in vivo* (39, 42) and in humans (43), respectively, FimH_DG_-Ferritin mRNA vaccination conferred greater protective efficacy in the bladder of UPEC-experienced mice at the doses tested. Together, these data demonstrate the potential of the FimH_DG_-Ferritin mRNA vaccine to provide protection in individuals with rUTIs.

The protective efficacy of a vaccine can be mediated by both humoral and cellular immune mechanisms, with humoral immune responses contributing through direct pathogen neutralisation as well as immune recruitment (44). To further understand the immunological basis underlying the observed protective efficacy, we therefore examined vaccine-induced FimH-specific binding and functional antibody responses, together with inflammatory and immune recruitment markers. A correlation analysis revealed significant relationships between vaccine group and serum HAI EC_90_ titres, as well as between serum HAI EC_90_ titres and bladder bacterial load; however, the association between HAI EC_90_ titres and bladder CFUs was no longer significant after adjustment for vaccine group. Taken together, these findings suggest that differences in bladder bacterial burden are primarily driven by vaccine-specific effects and indicate that functional serum antibody responses are associated with protective efficacy, although their independent contribution cannot be distinguished from other vaccine-induced immune mechanisms in the present study. While robust FimH-specific CD4^+^ and CD8^+^ T cell responses were observed in UPEC-naïve animals, the contribution of cellular immunity to protection was not directly evaluated in the rUTI challenge model and therefore remains to be determined. Consistently, FimH-specific IgG binding antibody levels in urine increased on average 15-fold following challenge infection, indicating increased bladder permeability or immune access. Previous observations in UPEC-naïve animals showed that IgG responses in the serum did not correlate with equivalent urine IgG levels (25), a pattern also observed here; however, the robust protective efficacy conferred in the bladder supports our hypothesis that serum-derived, FimH-specific functional IgG antibodies may represent a correlate of vaccine-induced protection, consistent with previous findings (41, 45). While mucosal immune responses, such as IgA, IgM, and/or tissue-resident B and T cells, may also contribute to protection (46, 47), our results suggest that infection-driven inflammation increases bladder permeability, facilitating immune access of vaccine-induced, anti-FimH IgG antibodies from the circulation. In addition to antibody-mediated effects, vaccination was associated with a significant reduction in G-CSF and CXCL1 following challenge infection, cytokines linked to increased pathogen virulence (48) and acute cystitis in elderly patients (49), respectively, indicating that vaccination with FimH_DG_-Ferritin may also protect against severe disease.

Increased protective efficacy may be driven by the improved immunogenicity of the FimH_DG_-Ferritin mRNA vaccine relative to protein subunit vaccine controls, previously demonstrated by administration of two FimH_DG_-Ferritin dose levels in UPEC-naïve rats (25). In the present study, we build on this work by evaluating five dose levels of the final vaccine construct, FimH_DG_-Ferritin with N1mΨ-modified nucleosides, in UPEC-naïve mice and rats. Immunogenicity was not assessed against protein subunit vaccine controls; however, robust binding and functional serum antibody titres elicited by FimH_DG_-Ferritin mRNA vaccination were within a similar range to titres from clinical observations reported in the development of other UPEC vaccines, suggesting translation potential (43, 50). Our previous proof-of-concept study harnessed the BAI assay to assess functional antibody response against one UPEC strain using within-group pooled samples (25). In the present study, we improve understanding of vaccine-induced immunity via assessment of multiple homologous and heterogenous UPEC strains, with functional antibody response measured in individual animal samples using two independent assays. Correlation analysis demonstrated that serum-derived inhibition of bacterial adhesion to human bladder cells in the BAI assay provided a reliable measure of antibody functionality. BAI and HAI assay performance were comparable, thereby independently validating functional antibody responses using a second method. Additionally, FimH-specific IgG binding antibodies were detected in urine, indicating access of humoral immune factors to the site of infection. Functional antibody responses were observed against multiple heterologous and homologous UPEC strains in this study, challenging the notion that genetic and antigenic diversity of UPEC is a barrier to the development of an effective vaccine (46), and supporting type 1 pili as highly expressed (51, 52), widely conserved (53), and potent attenuation targets of UPEC (54). Together, these data demonstrate that FimH_DG_-Ferritin mRNA immunisation can elicit strong immune responses in physiologically relevant tissues, with the potential for broad protection against UTI caused by diverse UPEC strains.

A central feature of the FimH_DG_-Ferritin mRNA vaccine design is the multimeric display of FimH on the surface of *H. pylori* ferritin PNPs, which form with functional, immune-accessible epitopes *in vitro*. Scaffold-based vaccines are adaptable platforms that are predicted to outperform traditional vaccine types, such as monomeric protein subunit vaccines, across multiple infectious diseases, including seasonal influenza (55), COVID-19 (56), and HIV (57). Importantly, *H. pylori* ferritin-based nanoparticle vaccines have shown a favourable safety profile in multiple human clinical trials (58, 59), and we observed no evidence of human ferritin cross-reactivity following repeated FimH_DG_-Ferritin mRNA vaccination in rodents.

Together, these findings demonstrate that FimH_DG_-Ferritin mRNA vaccination, as shown by its robust protective efficacy in a physiologically relevant rUTI mouse model, has the potential to address key challenges in UPEC vaccine development, including strain diversity, immune accessibility of the bladder, and the need for protection in individuals with recurrent infection (46, 60). This work significantly advances our previous study (25) by demonstrating protective efficacy in UPEC-experienced animals, while also providing detailed characterisation of FimH_DG_-Ferritin PNP formation and expanded evaluation of homologous and heterologous functional antibody responses. While optimisation of dosing regimens will be an important consideration in future clinical development, the ability of the FimH_DG_-Ferritin mRNA vaccine to confer protection in UPEC-experienced hosts underscores its promise as a next-generation vaccine candidate for the prevention of rUTIs.

## 5 Conclusion

By encoding multimeric FimH_DG_-Ferritin nanoparticles that elicit potent, broad-spectrum functional immune responses and confer protective efficacy *in vivo*, the FimH_DG_-Ferritin mRNA vaccine emerges as a compelling candidate for the prevention of UPEC-caused UTI, warranting evaluation in clinical studies.

## Data Availability

The original contributions presented in the study are included in the article/**Supplementary Material**, further inquiries can be directed to the corresponding author.
