# Supplementary material for: An mRNA-based FimH nanoparticle vaccine against uropathogenic Escherichia coli confers protection in a mouse model of recurrent urinary tract infection
Source: Front Immunol. 2026 Sep 3;17:1920497. doi: 10.3389/fimmu.2026.1920497 (PMC13582291; doi:10.3389/fimmu.2026.1920497)
Supplement: Supplementary file 1 [file DataSheet1.docx]

Supplementary Material

An mRNA-based FimH nanoparticle vaccine against uropathogenic Escherichia coli confers protection in a mouse model of recurrent urinary tract infection

**Supplementary Table 1.** FimH protein sequence differences between UPEC strains tested in HAI assays

**Supplementary Figure 1.** nPAGE reveals large FimH_DG_-Ferritin protein assemblies in cell culture supernatants and lysates

**Supplementary Figure 2.** PNGase F treatment indicates that the majority of secreted FimH_DG_-Ferritin fusion proteins are fully N-glycosylated in transfected HeLa cells

**Supplementary Figure 3.** Dose-dependent early innate immune responses upon IM administration with the FimH_DG_-Ferritin mRNA vaccine in UPEC-naïve animals

**Supplementary Figure 4.** Assessment of anti-ferritin binding IgG antibodies in serum of UPEC-naïve animals following 2 or 3 administrations of the FimH_DG_-Ferritin mRNA vaccine

**Supplementary Figure 5.** FimH_DG_-Ferritin mRNA vaccination induces dose-dependent functional antibody responses against the homologous UPEC strain in serum of UPEC-naïve animals as measured by the BAI

**Supplementary Figure 6.** Gating strategy for T cell analysis

**Supplementary Figure 7**. FimH_DG_-Ferritin mRNA vaccination induces serum anti-FimH IgG responses in a rUTI mouse model

**Supplementary Figure 8.** FimH_DG_-Ferritin mRNA vaccination reduces CXCL1 and G-CSF levels following UPEC challenge in a rUTI mouse model

**Supplementary Data:** Statistical Analysis Parameters, Results and Correlation Analysis (presented in a separate Excel file)

**Supplementary Table 1. FimH protein sequence differences between UPEC strains tested in HAI assays**

| **UPEC isolate** | **Multilocus**  **Sequence**  **Type^a^** | **NCBI *fimH***  **Sequence Link** | ***fimH***  **Sequence**  **Type^b^** | **FimH Protein Sequence**  **Differences Relative to UTI89** |
| --- | --- | --- | --- | --- |
| UTI89 | 95 | [UTI89 fimH](https://www.ncbi.nlm.nih.gov/nuccore/CP000243.1?from=4913555&to=4914457&report=fasta)  (GenBank:  CP000243.1) | *fimH*18 | - |
| CFT073 | 73 | [CFT073 fimH](https://www.ncbi.nlm.nih.gov/nuccore/FJ865781.1?report=fasta)  (GenBank:  AE014075.1) | *fimH*10 | A62S/V163A |
| J96 | 12 | [J96 fimH](https://www.ncbi.nlm.nih.gov/nuccore/FJ865781.1?report=fasta)  (GenBank:  FJ865781.1) | *fimH*27 | A27V/A62S/S70N/N78S |
| EC958 | 131 | [EC958 fimH](https://www.ncbi.nlm.nih.gov/nuccore/HG941718.1?from=5024352&to=5025254&report=fasta)  (GenBank:  HG941718.1) | *fimH*30 | A62S/S70N/N78S/R166H |

^a^MLST information (Achtman 7 Gene MLST) obtained from EnteroBase: <https://enterobase.warwick.ac.uk/species/index/ecoli>; ^b^*fimH* allele determined by fimTyper: <https://cge.food.dtu.dk/services/FimTyper/>

**Supplementary Figure 1. nPAGE reveals large FimH_DG_-Ferritin protein assemblies in cell culture supernatants and lysates**

nPAGE and western blot analyses indicating PNP formation of (**A**, **C**) non-tagged and (**B**, **C**) His-tagged versions of FimH_DG_-Ferritin in HeLa cell (**B**, **C**) lysates and (**A**-**C**) supernatants following (**A**) mannose-agarose affinity purification or (**B**) Ni-NTA purification. HeLa cells were transfected with (**A**) 20 μg LNP formulated FimH_DG_-Ferritin mRNA or (**B, C**) 40 μg FimH_DG_-Ferritin mRNA using Lipofectamine MessengerMax. Lysates and supernatants (SN) were collected 72 h post transfection, purified for (**A**) and (**B**), and analysed by nPAGE and western blot against FimH-specific monoclonal mouse antibody (54G9). The SN and lysate lanes in (**B**) and all samples in (**C**) represent unpurified samples. A native molecular weight marker was added to each gel and the molecular weight of each signal in kDa is indicated. Native molecular weight markers were stained with total protein stain and imaged prior to antibody incubation.

h, hours; kDa, kilodalton; nPAGE, native polyacrylamide gel electrophoresis; PNP, protein nanoparticle; SN, supernatant

**Supplementary Figure 2. PNGase F treatment indicates that the majority of secreted FimH_DG_-Ferritin fusion proteins are fully N-glycosylated in transfected HeLa cells**

Western blot of transfected HeLa cell lysate and supernatant following PNGase F treatment. HeLa cells were transfected with 40 μg FimH_DG_-Ferritin mRNA using Lipofectamine MessengerMax. Lysate and supernatant (SN) were collected 72 h post transfection, treated with PNGase F and analysed by western blot using FimH-specific monoclonal mouse antibody (54G9) for detection. The molecular weight (in kDa) of each signal is indicated.

h, hours; kDa, kilodalton; SN, supernatant

**Supplementary Figure 3. Dose-dependent early innate immune responses upon IM administration with the FimH_DG_-Ferritin mRNA vaccine in UPEC-naïve animals**

Cytokines and chemokines were measured in serum of UPEC-naïve animals in dose-response immunogenicity studies in (**A–F**) female BALB/c AnNRj mice (n=24/group) and (**G–I**) female Wistar rats (n=24/group) by CBA. Animals were injected IM with the FimH_DG_-Ferritin mRNA vaccine on Day 0 with FimH_DG_-Ferritin mRNA vaccine doses 0.04 μg, 0.2 μg, 1 μg and 5 μg (mice) or 0.08, 0.4, 2, 10 and 20 μg (rats). Control animals received 0.9% NaCl (n=12/group) via IM route. (**A–F**) Cytokine and chemokine levels were determined via CBA using the LEGENDplex Mouse Anti-Virus Response Panel (13-plex) in mouse serum samples collected 18 h after the first injection. The levels of (**A**) IFNα, (**B**) IL-6, (**C**) IFNγ, (**D**) CXCL1, (**E**) CCL2 and (**F**) CXCL10 are shown. (**G–I**) Cytokine and chemokine levels were determined via CBA using the LEGENDplex Rat Inflammation Panel (13-plex) in rat serum samples collected 18 h after the first vaccine or control administration. The levels of (**G**) IL-6, (**H**) CCL-2 and (**I**) IL-10 are shown. Each dot represents an individual animal, bars depict the mean, error bars depict the SD. Dotted lines represent the respective LLOQ. Values below the LLOQ were set to half of the respective LLOQ.

CBA, cytometric bead assay; h, hours; IM, intramuscular; LLOQ, lower limit of quantification; mRNA, messenger RNA; SD, standard deviation; UPEC, uropathogenic *E. coli*

**Supplementary Figure 4. Assessment of anti-ferritin binding IgG antibodies in serum of UPEC-naïve animals following 2 or 3 administrations of the FimH_DG_-Ferritin mRNA vaccine**

Ferritin-specific IgG binding antibodies were measured by ELISA in serum of UPEC-naïve animals in dose-response immunogenicity studies in (**A**, **C**, **E**) female BALB/c AnNRj mice (n=12/dose schedule) and (**B**, **D**, **F**) female Wistar rats (n=12/dose schedule). Animals received either 2 or 3 IM administrations of the FimH_DG_-Ferritin mRNA vaccine on Days 0, 28 (all) and 56 (3-dose only) at doses of 0.04, 0.2, 1 or 5 μg (mice) or 0.08, 0.4, 2, 10 and 20 μg (rats). Control animals received 0.9% NaCl (n=6/dose schedule). Anti-ferritin IgG endpoint titres against (**A**, **B**) *H. pylori* ferritin, (**C**, **D**) human ferritin light chain and (**E**, **F**) human ferritin heavy chain were measured in serum collected two weeks post second administration (Day 42, orange bars, 2-dose schedule only) or two weeks post third administration (Day 70, blue bars). Due to limited sample availability, sample sizes for specific groups were reduced as follows: (**E**) Day 42: 5 µg (n=11); (**E**) Day 70: NaCl (n=4); (**B**, **D**, **F**) Day 42: 0.4 µg (n=11), 10 µg (n=11). Each dot represents an individual animal, bars depict the geometric mean, error bars depict the geometric SD. The dotted line represents the LLOQ. Values below the LLOQ of 100 were set to 20. The statistical difference between 2- and 3-dose schedules across all dose levels was analysed using a two-way ANCOVA (***P*<0.01; ****P*<0.001; ns, not significant).

ANCOVA, Analysis of Covariance; ELISA, enzyme-linked immunosorbent assay; IM, intramuscular; LLOQ, lower limit of quantification; mRNA, messenger RNA; SD, standard deviation; UPEC, uropathogenic *E. coli*

**Supplementary Figure 5. FimH_DG_-Ferritin mRNA vaccination induces dose-dependent functional antibody responses against the homologous UPEC strain in serum of UPEC-naïve****animals as measured by the BAI assay**

FimH-specific functional antibodies were measured by BAI assay in serum of UPEC-naïve animals in dose-response immunogenicity studies in (**A, C**) female BALB/c AnNRj mice (n=12/dose schedule) and (**B, D**) female Wistar rats (n=12/dose schedule). Animals received either 2 or 3 IM administrations of the FimH_DG_-Ferritin mRNA vaccine on Days 0, 28 (all) and 56 (3-dose only) at doses of 0.04, 0.2, 1 or 5 μg (mice) or 0.08, 0.4, 2, 10 and 20 μg (rats). Control animals received 0.9% NaCl (n=6/dose schedule). BAI (**A**, **B**) EC_25_ and (**C**, **D**) EC_50_ antibody titres against the homologous UPEC strain J96 were measured in serum collected two weeks post second administration (Day 42, orange bars, 2-dose schedule only) or two weeks post third administration (Day 70, blue bars). BAI titres are reported as EC_25_ or EC_50_ values indicating the reciprocal serum dilution resulting in 25% or 50% reduction, respectively, in UPEC binding to human uroepithelial cells. Due to limited sample availability, sample sizes for specific groups were reduced as follows: (**A**) Day 42: 5 µg (n=11); Day 70: 1 µg (n=11), 5 µg (n=11); (**B**) Day 42: 0.4 µg (n=8), 10 µg (n=11); (**C**) Day 42: 5 µg (n=10); Day 70: 1 µg (n=9), 5 µg (n=11); (**D**) Day 42: 0.4 µg (n=9), 10 µg (n=11); Day 70: 10 µg (n=9). Each dot represents an individual animal, bars depict the geometric mean, error bars depict the geometric SD. The dotted line represents the LLOQ. Values below the LLOQ were set to half of the respective LLOQ. The statistical difference between 2- and 3-dose schedules across all dose levels was analysed using a two-way ANCOVA (ns: not significant).

ANCOVA, Analysis of Covariance; BAI, bacterial adhesion inhibition; IM, intramuscular; LLOQ, lower limit of quantification; mRNA, messenger RNA; SD, standard deviation; UPEC, uropathogenic *E. coli*

**Supplementary Figure 6. Gating strategy for T cell analysis**

FimH-specific T cell responses were measured in splenocytes of FimH_DG_-Ferritin mRNA-vaccinated animals as described in Figure 4. Isolated splenocytes were re-stimulated with a 15-mer peptide library spanning the FimH full length protein and FimH-specific CD8^+^ and CD4^+^ T cell responses were analysed in mouse samples by ICS and flow cytometry. The flow blots shown illustrate the gating strategy using splenocytes isolated from a mouse vaccinated with the FimH_DG_-Ferritin mRNA vaccine as an example. Splenocytes were first gated on singlets to exclude doublets, followed by selection of viable cells to remove dead cells. From the viable singlet population, T cells were identified as Thy1.2⁺ cells. Within the Thy1.2⁺ T cell compartment, CD4⁺ T cells were defined as CD4⁺/CD8⁻ events and CD8⁺ T cells as CD8⁺/CD4⁻ events. CD4⁺ and CD8⁺ T cell populations were then further subdivided into multifunctional subsets based on intracellular expression of IFN‑γ and TNF.

CD, cluster of differentiation; ICS, intracellular cytokine staining; mRNA, messenger RNA; SD, standard deviation TNF, tumour necrosis factor

**B**

**A**

**Supplementary Figure 7. FimH_DG_-Ferritin mRNA vaccination induces serum anti-FimH IgG responses in a rUTI mouse model**

Pre-challenge FimH-specific IgG binding antibodies in serum were measured by ELISA in the rUTI mouse model using C3H/HeN mice. Sera were collected two weeks after the second or third immunisation (two weeks prior to challenge infection). Sample sizes (n) for the 2-dose schedule were: NaCl, 13; irrelevant mRNA, 13; FimHC-Freund’s, 12; FimHC-PHAD, 10; FimH_DG_-Ferritin 0.2 µg, 13; FimH_DG_-Ferritin 5 µg, 13. For the 3-dose schedule, n values were: NaCl, 13; irrelevant mRNA, 13; FimHC-Freund’s, 12; FimHC-PHAD, 12; FimH_DG_-Ferritin 0.2 µg, 13; FimH_DG_-Ferritin 5 µg, 13. Each dot represents an individual animal (geometric mean of two replicates), bars depict the geometric mean, error bars depict the geometric SD. The dotted line represents the LLOQ. Values below the LLOQ (100) were set to 20 for each individual replicate. Statistically significant differences between the treatment groups and the NaCl control group were determined by Kruskal-Wallis test with Dunn’s multiple comparison post hoc test (**P* <0.05; ****P* <0.001; *****P* <0.0001; ns: not significant).

ELISA, enzyme-linked immunosorbent assay; LLOQ, lower limit of quantification; mRNA, messenger RNA; PHAD, Phosphorylated HexaAcyl Disaccharide; rUTI, recurrent urinary tract infection; SD, standard variation; UPEC, uropathogenic *E. coli*

**Supplementary Figure 8. FimH_DG_-Ferritin mRNA vaccination reduces CXCL1 and G-CSF levels following UPEC challenge in a rUTI mouse model**

The concentration of (**A**) CXCL1 in post-challenge bladder homogenate samples as well as (**B**) CXCL1 and (**C**) G-CSF in post-challenge serum samples was assessed by ELISA in the rUTI mouse model using C3H/HeN mice. Samples were collected 24 h post challenge on Day 99 (2-dose schedule) or on Day 127 (3-dose schedule). (**A**, **B**, **C**) Sample sizes (n) for the 2-dose schedule were: NaCl, 13; irrelevant mRNA, 13; FimHC-Freund’s, 12; FimHC-PHAD, 8; FimH_DG_-Ferritin 0.2 µg, 13; FimH_DG_-Ferritin 5 µg, 13. For the 3-dose schedule, n values were: NaCl, 13; irrelevant mRNA, 13; FimHC-Freund’s, 11; FimHC-PHAD, 12; FimH_DG_-Ferritin 0.2 µg, 13; FimH_DG_-Ferritin 5 µg, 13. Each dot represents an individual animal, bars depict the mean, error bars depict the SD. Dotted lines represent the respective LLOQ. Statistically significant differences between the treatment groups and the NaCl control group were determined by one-way ANOVA with Dunnett’s multiple comparison post hoc test of log_10_-transformed data (**P* <0.05; ***P* <0.01; ****P* <0.001; *****P* <0.0001; ns: not significant).

ANOVA, Analysis of Variance; ELISA, h, hours; IM, intramuscular; LLOQ, lower limit of quantification; mRNA, messenger RNA; PHAD, Phosphorylated HexaAcyl Disaccharide; rUTI, recurrent urinary tract infection; SD, standard deviation; UPEC, uropathogenic *E. coli*
